# Higher Memory Responses in HIV-Infected and Kidney Transplanted Patients than in Healthy Subjects following Priming with the Pandemic Vaccine

**DOI:** 10.1371/journal.pone.0040428

**Published:** 2012-07-27

**Authors:** Claire-Anne Siegrist, Christian van Delden, Michael Bel, Christophe Combescure, Cécile Delhumeau, Matthias Cavassini, Olivier Clerc, Sara Meier, Karine Hadaya, Paola M. Soccal, Sabine Yerly, Laurent Kaiser, Bernard Hirschel, Alexandra Calmy, H1N1 Study Group, Swiss HIV Cohort Study (SHCS)

**Affiliations:** 1 Department of Pathology-Immunology and Paediatrics, Centre for Vaccinology, University Hospitals of Geneva and Faculty of Medicine, University of Geneva, Geneva, Switzerland; 2 Service of Transplantation, Department of Surgery, University Hospitals of Geneva and Faculty of Medicine, University of Geneva, Geneva, Switzerland; 3 Clinical Research Centre, University Hospitals of Geneva and Faculty of Medicine, University of Geneva, Geneva, Switzerland; 4 Service of Infectious Diseases, Centre Hospitalier Universitaire Vaudois, Lausanne, Switzerland; 5 Laboratory of Virology and Swiss National Centre for Influenza, Department of Genetics and Laboratory Medicine and Department of Medical Specialities, University Hospitals of Geneva and Faculty of Medicine, University of Geneva, Geneva, Switzerland; 6 HIV Unit, Division of Infectious Diseases, Department of Internal Medicine, University Hospitals of Geneva and Faculty of Medicine, University of Geneva, Geneva, Switzerland; 7 Division of Infectious Diseases, University of Lausanne, University Hospital, Lausanne, Switzerland; University of Pittsburgh, United States of America

## Abstract

**Background:**

Memory responses require immune competence. We assessed the influence of priming with AS03-adjuvanted pandemic vaccine (Pandemrix®) on memory responses of HIV patients, kidney recipients (SOT) and healthy controls (HC).

**Method:**

Participants (HIV: 197, SOT: 53; HC: 156) were enrolled in a prospective study and 390/406 (96%) completed it. All had been primed in 2009/2010 with 1 (HC) or 2 (patients) doses of Pandemrix®, and were boosted with the 2010/2011 seasonal influenza vaccine. Geometric mean titres and seroprotection rates were measured 12 months after priming and 4 weeks after boosting. Primary and memory responses were directly compared in 191 participants (HCW: 69, HIV: 71, SOT: 51) followed during 2 consecutive seasons.

**Results:**

Most participants (HC: 77.8%, HIV: 77.6%, SOT: 66%) remained seroprotected at 12 months post-priming. Persisting A/09/H1N1 titers were high in HIV (100.2) and HC (120.1), but lower in SOT (61.4) patients. Memory responses reached higher titers in HIV (507.8) than in HC (253.5) and SOT (136.9) patients. Increasing age and lack of HAART reduced persisting and memory responses, mainly influenced by residual antibody titers. Comparing 2009/2010 and 2010/2011 titers in 191 participants followed for 2 seasons indicated lower post-2010/2011 titers in HC (240.2 vs 313.9), but higher titers in HIV (435.7 vs 338.0) and SOT (136 vs 90.3) patients.

**Conclusions:**

Priming with 2 doses of Pandemrix® elicited persistent antibody responses and even stronger memory responses to non-adjuvanted seasonal vaccine in HIV patients than 1 dose in healthy subjects. Adjuvanted influenza vaccines may improve memory responses of immunocompromised patients.

**Trial Registration:**

ClinicalTrials.gov NCT01022905

## Introduction

Immunosuppressed patients are at higher risks of influenza complications. This was evidenced by reports of disease outcome in solid organ transplant (SOT) recipients infected by the pandemic influenza A/09/H1N1 strain [1]–[6]. A similar risk exists in HIV infected individuals with advanced disease and low CD4 cell count but not in HAART-treated patients [7]–[11].

Immunosuppressed patients have a general trend toward impaired antibody responses to non-adjuvanted vaccines [6]. Short-term antibody responses were indeed lower following 1 or 2 doses of non-adjuvanted influenza A/09/H1N1 vaccines in HIV-infected [12], [13] and solid organ transplant (SOT) patients [13]–[15]. The extent to which adjuvanted vaccines may improve responses is thus of central interest. In HIV-infected patients, a single dose of the AS03-adjuvanted pandemic vaccine (Pandemrix®) elicited higher responses than non-adjuvanted monovalent vaccines [16]. Four weeks after 1 dose of Pandemrix®, seroresponses remained lower than in controls [17], [18] reaching similar titers after 2 doses [19]–[21]. Seroresponses remained lower in SOT recipients even after 2 doses of Pandemrix® [19], [22], [23], reflecting a more profound impact of immunosuppression on vaccine responses.

How immunosuppression affects memory responses is less well defined. In HIV-infected patients, impaired B and T cell functions cause dysfunctional germinal center interactions [24] and result in a progressive loss of B-cell memory despite antiretroviral therapy [25]–[27]. Accordingly, most HAART-treated HIV-infected adults reached a hemagglutination inhibition titer (HAI) ≥1/40 four weeks after immunization with non-adjuvanted A/09/H1N1 vaccines but only 28% remained above this threshold at 6 months [28]. How the immunosuppression of SOT patients affects memory responses is less well described.

In 2009/2010, we had followed 760 immunocompromised and 133 healthy adults immunized with 1 (healthy) or 2 (patients) doses of Pandemrix®. Four weeks after immunization, we observed similar responses in HIV-infected individuals after 2 doses as in healthy adults after 1 dose [20], and lower seroresponses in SOT recipients despite 2 immunizations [23]. To define how adjuvanted vaccines would influence antibody persistence and memory responses, we assessed the impact of 2009/2010 priming with Pandemrix® on antibody persistence and memory responses elicited in 2010/2011 by one dose of a non-adjuvanted trivalent inactivated seasonal vaccine including the same influenza A/09/H1N1 strain.

## Patients and Methods

### Subjects

This prospective, multisite, open-label study recruited 406 subjects in November 2010∶197 HIV-infected patients, 53 SOT (kidney) recipients and 156 healthy controls (HC) (Figure S1). Inclusion criteria included age above 18 years and having received 1 (controls) or 2 (patients) doses of AS03-adjuvanted pandemic influenza vaccine in 2009/2010. Among these 406 subjects, 191 subjects (69 HIV-infected patients, 51 kidney transplant recipients and 71 controls) had already been enrolled in 2009/2010 and were followed for 2 seasons. The latter are referred to as the 2009–2010–2011 cohort. The study was approved by the institutional review board (ID: CER-09-234), registered on ClinicalTrials.gov prior to patient enrolment (ID: NCT01022905) and conducted in accordance with the principles of the Declaration of Helsinki, the standards of Good Clinical Practice, and Swiss regulatory requirements. Written informed consent was obtained prior to inclusion in 2009 and in 2010.

The protocol for this trial is available as supporting information, see Protocol S1.

### Vaccines and Immunizations

In 2010/2011, patients and controls received one intramuscular dose of a non-adjuvanted trivalent split-virus influenza vaccine containing 15 µg of A/California/07/2009 (H1N1), A/Perth/16/2009 (H3N2) and B/Brisbane/60/2008. Most patients (>98% in each group) received Mutagrip® (SanofiPasteurMSD). In 2009/2010, all participants had received 1 (healthy controls) or 2 doses (HIV-infected or SOT patients) of AS03-adjuvanted split-virus influenza A/09/H1N1 vaccine (Pandemrix®, GlaxoSmithKline) at a 4 weeks interval, according to official Swiss recommendations. Each dose of Pandemrix® contained 3.75 µg of A/09/H1N1 antigen emulsified in squalene, DL-á-tocopherol and polysorbate 80.

### Data Collection

Medical information was obtained through a detailed medical history and completed through the patient’s records. Blood was collected on the day of the first dose and 4 weeks after immunization. Sera were prepared and stored at -20°C until used.

### Haemagglutination Inhibition (HAI) Assay

HAI assays were performed as described [29]. Sera were subjected to 2-fold serial dilutions prior to incubation with 4 or 8 HA units of pandemic influenza A/California/7/09 (H1N1) and seasonal A/Perth/09 (H3N2) virus, respectively. Results were expressed as the reciprocal of the highest dilution showing a positive HAI. Negative samples were assigned a titre of 1∶4 and individual values were log transformed to calculate the geometric mean antibody titres (GMT).

The three co-primary immunogenicity end-points were: 1) The GMTs based on individual HAI titres, 2) the proportion of seroprotected subjects (defined as a post-vaccination HAI-titre of ≥1∶40) and 3) the proportion of subjects with a seroresponse (defined as a post-vaccination HAI-titre of ≥1∶40 and a fold increase in GMT of ≥4 between pre-vaccination and post-vaccination HAI- titres).

### Safety Monitoring

Safety endpoints were divided into local inflammation (injection-site pain, erythema and swelling) and systemic reactions (fever, fatigue, headaches and anorexia). They were self-recorded for 7 days after each dose. Serious adverse events (SAE) were actively searched for and followed up until their resolution; recording stopped on February 28, 2010, at study termination. In HIV-infected patients, HIV-RNA levels were measured 4 weeks after immunization to detect potential increases in HIV-RNA levels. Quantitative plasma HIV-1 RNA was measured on a Roche COBAS TaqMan HIV-1 test version 2.0 (Roche Diagnostic, Basel, Switzerland). Transplant patients were closely followed and instructed to contact the study investigator in case of adverse events.

### Statistical Analysis

Due to the lack of data concerning the long-term immunogenicity of Pandemrix® at time of study design, sample size was based on recruitment capacity. GMTs were given with 95% confidence interval. The reverse cumulative distributions were presented. The comparison of titres between individual strata was assessed by means of the Kruskal-Wallis test. For longitudinal comparisons, the Wilcoxon test for paired data was used (for instance baseline vs post-baseline titers from same patients). Comparisons of proportions were performed by using Chi-squared tests (or Fisher exact test if frequencies were less than 5) and McNemar tests for paired data. Multivariate regression models were constructed to investigate the association between specific independent variables and post-vaccination antibody titres. The selection of factors introduced in the models was determined a priori. Data were logarithmically transformed prior to analysis. The parameter of the linear model indicated the variation on the log-transformed titre and we interpreted the meaning of the regression parameters as the percentage increase of the titre per given unit (for continuous factors) or compared to the reference category (for categorical factors). A logistic regression was performed to assess the association of the gender on the risk of pain adjusted on the group of patients. An odds ratio greater than 1 was interpreted as an increase of risk in women compared to men. The significance level was defined as 0.05. Data were analysed by using S-PLUS 8.0, Insightful Corp., Seattle, WA (USA).

## Results

### Characteristics of Immunosuppressed Patients and Healthy Subjects

The clinical characteristics of the 197 HIV-infected patients, 53 SOT recipients and 156 healthy controls recruited in 2010/2011 are described in Table 1. All had been immunized with Pandemrix® in 2009/2010. HCs included a higher percentage of females and were younger. Most participants were Caucasians (82%) and had been immunized against seasonal influenza in 2009. HIV-infected patients were characterized by their CDC category, CD4 T cell count and nadir (**Table 1**). Most were treated with antiretroviral drugs. Kidney transplant recipients had reduced creatinine clearance (median 52, IQR 38–60). Most were on maintenance mycophenolate acid, tacrolimus and low dose steroids (**Table 1**). All had been transplanted more than one year ago (median 9.1 years, IQR 4.8–13.4), minimum 1.3 year). Two had been treated for a rejection episode within 12 months (40 and 351 days before immunization).

**Table 1:** Baseline characteristics.

		Controls		HIV		Kidney recipient		
Total	N	156		197		53		p value*
Women	N (%)	106	67.90%	62	31.50%	20	37.70%	
Median age, years (IQR)		42.9 (33.8–51.7)		47.6 (40.9–55.3)		61.6 (55.6–69.7)		<0.001
	<40 yrs	61	39.10%	44	22.30%	3	5.70%	<0.001
	40–60 yrs	84	53.80%	123	62.40%	21	39.60%	
	>60 yrs	11	7.10%	30	15.20%	29	54.70%	
Seasonal influenza 2009	N (%)	148	94.90%	183	92.90%	46	86.80%	0.14
Ethnicity, N (%)	Caucasian	148	94.9%	129	65.5%	47	88.7%	
	Afri. Sub-saharian	4	2.6%	43	21.8%	3	5.7%	
	Others	4	2.6%	25	12.7%	3	5.7%	
CDC category	A, N (%)			89	45.40%			
	B, N (%)			51	26.00%			
	C, N (%)			56	28.60%			
	Missing data			1	0.50%			
Antiretroviral therapy	None			11	5.60%			
	Including PI, no NNRTI			60	30.50%			
	Including NNRTI, no PI			84	42.60%			
	ART+PI+NNRTI			20	10.20%			
	HAART + other			22	11.20%			
CD4 Baseline, cells/mm3	Median (IQR)			615 (458–769)				
	Missing data			5	2.50%			
Nadir, cells/mm3	Median (IQR)			172 (50–288)				
	Missing data			1	0.50%			
Time since transplant, years	Median (IQR)					9.1 (4.8–13.4)		
Rejection episodes within	No					51	96.20%	
previous 12 months, N(%)	Yes					2	3.80%	
Immunosuppressive	Cyclosporin, N(%)					11	20.80%	
treatment	Tacrolimus, N (%)					37	69.80%	
	Evero- or Sirolimus, N (%)					2	3.80%	
	MMF or EC-MPA, N (%)					35	66.00%	
	Azathioprine, N (%)					8	15.10%	
	Others, N(%)					1	1.90%	
Oral steroids	N(%)					28	52.80%	
Low oral dose (<10 mg)	N(%)					25	89.30%	

* compared to controls.

IQR: interquartile range; PI: protease inhibitor; NNRTI : Non-Nucleoside Reverse Transcriptase Inhibitors; ART : antiretroviral therapy;

HAART: highly active antiretroviral therapy; MMF: mycophenolate mofetil; EC-MPA: enteric-coated mycophenolic acid.

All participants but one HC (newly recognized pregnancy) received a single dose of a non-adjuvanted trivalent inactivated 2010–2011 seasonal influenza vaccine, mostly Mutagrip®, allowing to directly compare their 2010–2011 responses despite differences in their 2009 immunization history. Seven HIV-positive patients and 1 SOT recipient remained unreachable. One SOT patient died of a non-vaccine related event. Six HCs withdrew consent to the 2^nd^ blood sample. Altogether, 190/197 (96.4%) of HIV patients, 51/53 (96.2%) of SOT recipients and 149/156 (95.5%) healthy subjects were included in the 2010/2011 analysis of vaccine responses (**Figure S1**).

### Safety

Immunization was generally well tolerated (**Table S1**). Local pain was more frequently reported by HCs (59.7% (CI95% 51.4; 67.7)) than HIV (42.1 (CI95% 35; 49.5)) or SOT (35.3 (CI95% 22.4; 49.9), p<0.001) patients. Women reported pain more frequently than men (OR adjusted on group: 2.06, CI95% 1.34–3.17), p<0.001) and, despite the higher proportion of women among HCs, the group effects remained significant: OR = 0.62 (CI95% 0.39–0.98,p = 0.04) for HIV compared to HCs and OR = 0.43 (CI95% 0.22–0.85, p = 0.02) for kidney recipients compared to HCs. Systemic reactions were rare and evenly distributed. Four weeks after immunization, HIV-RNA were below detection levels (172/197, 93%) or low (median 136 copies/mL, IQR 58–3048). SOT patients did not require any change of their immunosuppression regimen. Two severe adverse events (1 death, 1 amputation) were considered as unrelated to immunization.

### Antibody Persistence 12 Months after Monovalent AS03-adjuvanted Influenza A/09/H1N1 Immunization

**Figure 1:**
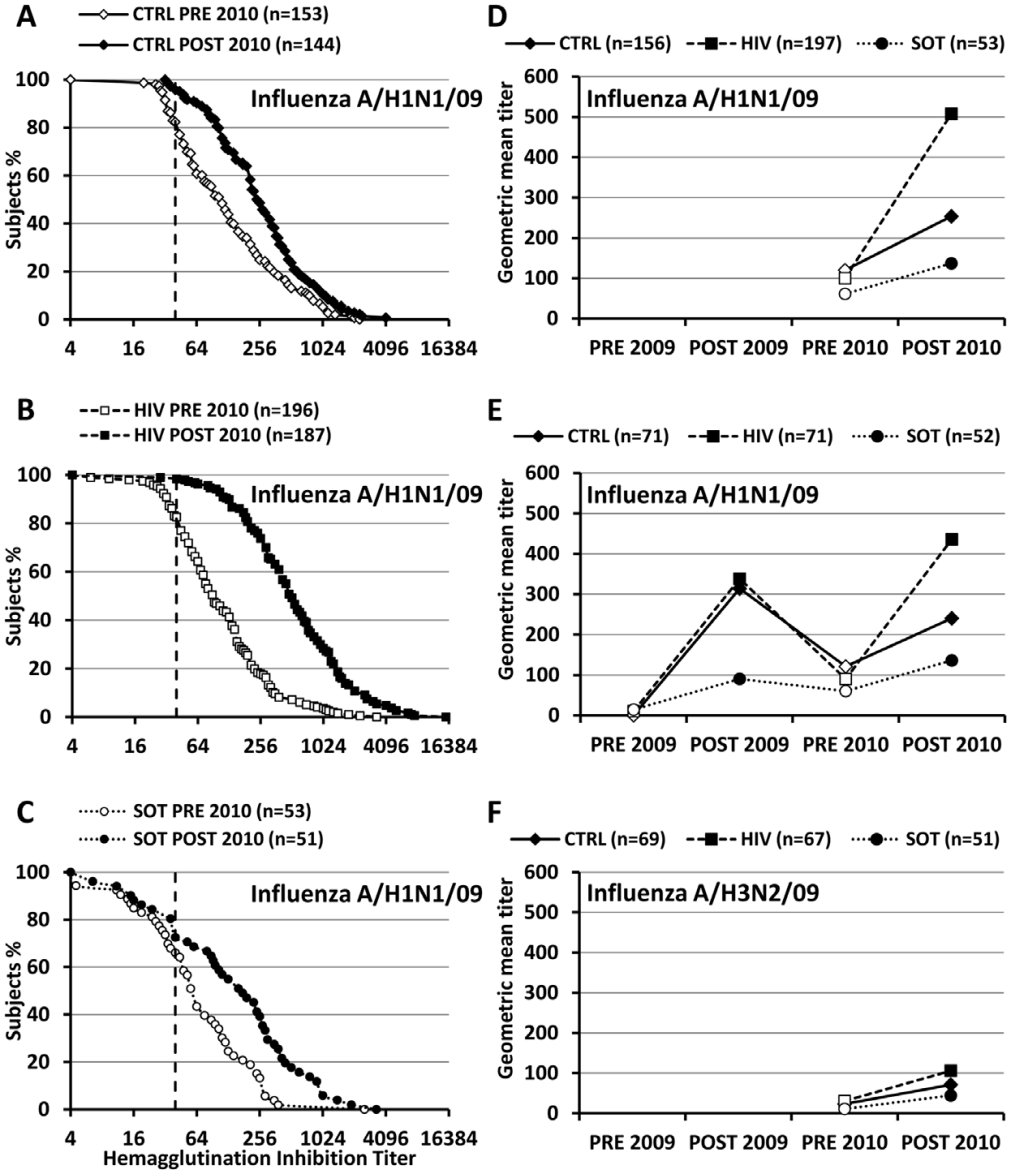
Distribution and kinetics of antibody titers. A–C. Blood was collected before and 4 weeks after 1 dose of non-adjuvanted 2010/2011 seasonal influenza vaccine. Antibody titers were assessed by hemagglutination inhibition (HAI). The results were expressed as the reciprocal of the highest dilution showing a positive HAI (see Methods). The vertical dotted line represents the seroprotection threshold (titer 1∶40). The curves represent the distribution of individual antibody titers in each group. D-F: Geometric mean HAI titers to influenza A/09/H1N1 (D, E) and A/09/H3N2 (F) were measured before (pre-2009) and after (post-2009) immunization with 1 (controls) or 2 (patients) doses of AS03-adjuvanted pandemic vaccine, and before (pre-2010) and after (post-2010) 1 dose of non-adjuvanted 2010/2011 seasonal influenza vaccine. D: All patients followed in 2010/2011. E -F: Patients followed for 2 consecutive seasons.

Antibody titers persisting 12 months after Pandemrix® immunization were assessed by HAI in 153/156 (98.0%) HCs, 196/197 (99.5%) HIV patients and all (53) kidney recipients. Most participants (HIV: 77.6%; HC: 77.8%; SOT 66%) remained seroprotected. Residual HAI-GMTs (pre-2010) were as high in HIV patients (100.2, CI95% 86.3–116.2) as in HCs (120.1, CI95% 99.7–144.6). Despite 2 doses of Pandemrix®, titers were lower in SOT recipients (61.4, CI95% 44.0-85.7) than in HIV patients or controls (**Table 2A** and **Figure 1** **A–B**).

**Table 2:** Antibody persistence and memory responses to influenza A/09/H1N1 and A/09/H3N2.

Influenza A/09/H1N1		HAI-GMT		Seroprotection		Seroconversion	
A. 2010/2011 cohort		N	(95%CI)	N	(%)	N/Total	(%)
**Controls**	Pre 2010	153	120.1 (99.7;144.6)	119	77.8 (70.4;84.1)		
	Post 2010	144	253.5 (213.4;301.1)	138	95.8 (91.2;98.5)	66/142	46.5 (38.1;55.0)
**HIV**	Pre 2010	196	100.2 (86.3;116.2)	152	77.6 (71.1;83.2)		
	Post 2010	187	507.8 (428.7;601.4)***	184	98.4 (95.4;99.7)	148/186	79.6 (73.1;85.1) ***
**Kidney recipients**	Pre 2010	53	61.4 (44;85.7) **	35	66 (51.7;78.5)		
	Post 2010	51	136.9 (89.7;209.1)*	37	72.5 (58.3;84.1) ***	20/51	39.2 (25.8;53.9)
**B. 2009/2010/2011 cohort**							
**Controls**	Post 2009	69	313.9 (218.6;450.8)	60	88.2 (78.1;94.8)		
	Pre 2010	69	121.1 (93.0;157.6)	55	79.7 (68.3;88.4)		
	Post 2010	69	240.2 (188.4;306.1)	67	97.1 (89.9;99.6)	27/67	40.3 (28.5;53.0)
**HIV**	Post 2009	66	338.0 (252.8;451.9)	62	93.9 (85.2;98.3)		
	Pre 2010	70	90.4 (70.1;116.6)	55	78.6 (67.1;87.5)		
	Post 2010	68	435.7 (320.8;591.9)***	65	95.6 (87.6;99.1)	49/67	73.1 (60.9;83.2) ***
**Kidney recipients**	Post 2009	52	90.3 (58.9;138.2) ***	35	67.3 (52.9;79.7)		
	Pre 2010	52	60.2 (42.9;84.4) **	34	65.4 (50.9;78.0)		
	Post 2010	50	136.0 (88.3;209.4)*	36	72.0 (57.5;83.8) ***	20/50	40 (26.4;54.8)

Influenza A/09/H3N2		HAI-GMT		Seroprotection		Seroconversion	
C. 2009/2010/2011 cohort		N	(95%CI)	N	(%)	N/Total	(%)
**Controls**	Pre 2010	69	23.2 (16.5;32.6)	30	43.5 (31.6;56.0)		
	Post 2010	69	71.1 (51.1;98.9)	48	69.6 (57.3;80.1)	31/69	44.9 (32.9;57.4)
**HIV**	Pre 2010	67	30.2 (21.9;41.7)	29	43.3 (31.2;56.0)		
	Post 2010	67	105.8 (74.5;150.3)	54	80.6 (69.1;89.2)	29/67	43.3 (31.2;56.0)
**Kidney recipients**	Pre 2010	51	10.7 (7.5;15.4)**	10	19.6 (9.8;33.1) **		
	Post 2010	51	44.2 (28.5;68.7)	30	58.8 (44.2;72.4)	22/51	43.1 (29.3;57.8)

HAI : hemagglutination inhibition; 2010/2011 cohort: subjects followed in 2010/2011; 2009/2010/2011 cohort: subjects followed for 2 years.

Pre 2010: before 2010/2011 immunization; Post 2010∶4 weeks after 2010/2011 immunization; Post 2009∶4 weeks after 1.

* p<0.05, **p<0.01 and *** p<0.001 compared to controls.

Regression analyses indicated that increasing age significantly lowered antibody persistence, reflected by lower pre-2010 HAI-GMTs (**Table 3**) and seroprotection rates (HC >60 years: 36.4% (CI95% 10.9-69.2) vs 81.0% (CI95% 73.6-87.1) for subjects ≤60 years, p = 0.003; HIV >60 years: 63.3% (CI95% 43.9-80.1) vs 80.1% (CI95% 73.2-85.9) for subjects ≤60 years, p = 0.07). Multivariate analyses (**Table 4**) confirmed that each additional 10 years reduced pre-2010 HAI-GMTs, although not significantly in HIV patients. Antibodies persisted at higher titers in HIV patients with CD4 counts >350/mm3 or HAART treated (**Table 4**). The immunosuppression regimens were too homogenous to lead to significant drug-specific influences (**Table 3** **and** **4**). Gender and 2009/2010 seasonal influenza immunization exerted no impact (not shown).

**Table 3:** Determinants of vaccine antibody persistence and memory responses to influenza A/09/H1N1.

			N	Pre 2010 HAI-GMT (95%CI)	Post 2010 HAI-GMT (95%CI)
**Controls**	*Age*	<40 yrs	61	166.6 (120.2;230.8)	335.4 (258.4;435.2)
		40-60 yrs	84	107.5 (85.7;134.9)	239.1 (190.8;299.5)
		>60 yrs	11	46.1 (31.2;68.2)	107.0 (54.0;212.0)
		*p-value*		0.001	0.005
	*Pre 2010 HAI-GMT*	*< median*	74	44.8 (40.3;49.9)	136.3 (111.0;167.3)
		≥ median	79	302.3 (251.7;363.0)	454.7 (373.2;554.1)
		*p-value*		<0.001	<0.001
**HIV-infected** **patients**	*Age*	<40 yrs	44	98.7 (73.6;132.3)	634.6 (469.4;857.9)
		40-60 yrs	123	107 (88.6;129.1)	516.5 (416.5;640.6)
		>60 yrs	30	78.1 (51.3;118.7)	353.2 (219.4;568.5)
		*p-value*		0.18	0.13
	*CD4 T cell count*	*> = 350*	163	110.5 (94.1;129.8)	518.2 (435.1;617.2)
		*<350*	29	57.8 (38.8;86.0)	477.7 (259.9;878.2)
		*p-value*		0.01	0.61
	*CD4 nadir*	*> = 100*	131	106.5 (88.8;127.8)	460.6 (381.3;556.4)
		*<100*	65	88.4 (68.0;114.9)	617.9 (441.2;865.3)
		*p-value*		0.29	0.02
	*CDC group*	*A*	89	93.1 (74.6;116.2)	421.0 (336.1;527.4)
		*B*	51	130.5 (98.0;173.7)	515.6 (387.6;685.8)
		*C*	56	88.3 (66.7;117.0)	667.6 (454.2;981.2)
		*p-value*		0.12	0.04
	*HAART*	*None*	11	42.4 (28.5;63.0)	212.5 (109.1;413.9)
		*ART+NNRTI+PI*	20	97.3 (59.4;159.4)	628.4 (408.7;966.4)
		*ART+NNRTI no PI*	84	108.3 (86.2;136.0)	463.7 (364.0;590.7)
		*ART+PI no NNRTI*	60	102.1 (77.3;134.7)	641.3 (450.7;912.6)
		*ART+Other*	22	111.8 (74.7;167.4)	501.3 (318.0;790.1)
		*p-value*		0.07	0.02
	*Pre 2010 HAI-GMT*	*< median*	96	43.0 (38.7;47.7)	336.9 (261.3;434.3)
		≥ median	100	225.7 (193.8;262.9)	754.3 (618.6;919.8)
		*p-value*		<0.001	<0.001
**Kidney** **recipients**	*Age*	<40 yrs	3	263.8 (27.1;2568.0)	368.0 (34.6;3918.5)
		40-60 yrs	21	69.1 (41.1;116.3)	130.5 (77.0;221.0)
		>60 yrs	29	48.5 (32.5;72.4)	127.4 (66.7;243.6)
		*p-value*		0.16	0.63
	*Tacrolimus*	*No*	16	44.5 (22.3;88.6)	178.3 (75.6;420.1)
		*Yes*	37	70.6 (48.7;102.3)	121.4 (75.1;196.2)
		*p-value*		0.27	0.20
	*MMF*	*No*	18	52.6 (30.2;91.8)	198.1 (96.0;408.9)
	*or EC-MPA*	*Yes*	35	66.5 (43.7;101.2)	113.9 (67.8;191.1)
		*p-value*		0.78	0.17
	*Oral steroids*	*No*	25	59.5 (33.4;105.9)	95.4 (49.4;183.9)
		*Yes*	28	63.2 (43.4;920)	188.9 (111.4;320.4)
		*p-value*		0.85	0.17
	*Pre 2010 HAI-GMT*	*< median*	25	22.8 (16.7;31.2)	57.2 (32;102.2)
		≥ median	28	148.6 (109.4;201.8)	297.7 (191.1;463.8)
		*p-value*		<0.001	<0.001

Continuous variable were tested as categorical variable based on biological cut-off or on medians.

HAI : hemagglutination inhibition; 95%CI: 95% Confidence Interval; Pre 2010: before 2010/2011 immunization;

Post 2010∶4 weeks after 2010/2011 immunization; PI: protease inhibitor; NNRTI : Non-Nucleoside Reverse.

Transcriptase Inhibitors; ART : antiretroviral therapy; HAART: highly active antiretroviral therapy;

MMF: mycophenolate mofetil; EC-MPA: enteric-coated mycophenolic acid.

**Table 4:** Multivariate regression analyses of anti-influenza A/09/H1N1 HAI titre determinants.

A. Healthy subjects	Multivariate analyses		
Pre 2010 HAI-GMTs	Estimate (se)	Relative change	p
*Age (per 10 years)*	-0.28 (0.08)	-25%	<0.001
Post 2010 HAI-GMTs			
*Age (per 10 years)*	-0.23 (0.08)	-20%	0.005
**B. HIV**			
Pre 2010 HAI-GMTs			
*Age (per 10 years)*	-0.06 (0.08)	-5%	0.50
*CD4> = 350*	0.63 (0.24)	88%	0.008
*Nadir CD4> = 100*	0.03 (0.23)	3%	0.91
*CDC A*			
*CDC B*	0.29 (0.20)	34%	0.15
*CDC C*	0.07 (0.25)	7%	0.77
*ART (compared to no ART)*	0.87 (0.34)	139%	0.01
Post 2010 HAI-GMTs			
*Age (per 10 years)*	-0.24 (0.09)	-22%	0.006
*CD4> = 350*	0.25 (0.26)	29%	0.34
*Nadir CD4> = 100*	-0.23 (0.25)	-21%	0.36
*CDC A*			
*CDC B*	0.18 (0.22)	19%	0.43
*CDC C*	0.40 (0.27)	49%	0.14
*ART (compared to no ART)*	0.92 (0.37)	151%	0.01
**C. Kidney recipients**			
Pre 2010 HAI-GMTs			
*Age (per 10 years)*	-0.43 (0.14)	-35%	0.003
*MMF or EC-MPA*	0.16 (0.36)	18%	0.65
*Tacrolimus*	0.40 (0.36)	48%	0.28
Post 2010 HAI-GMTs			
*Age (per 10 years)*	-0.34 (0.18)	-29%	0.06
*MMF or EC-MPA*	-0.55 (0.46)	-42%	0.24
*Tacrolimus*	-0.22 (0.47)	-20%	0.64

HAI : hemagglutination inhibition; se: standard error;

* Antibody titers were assessed before (Pre 2010) or 4 weeks after (Post 2010) 2010/2011 immunization.

### Memory Responses to Influenza A/09/H1N1 Immunization during the 2010/2011 Season

Four weeks after boosting with 1 dose of non-adjuvanted inactivated 2010–2011 trivalent vaccine including again the influenza A/09/H1N1 strain, HAI-GMTs had significantly increased in each group (**Figure 1A–C**). HCs raised strong responses (fold increase 2.07 (CI95% 1.81-2.37), p<0.001) and reached higher seroprotection rates (95.8 (CI95% 91.2-98.5) vs 77.8 (CI95% 70.4-84.1), p<0.001) compared to baseline (**Figure 1A** **and** **Table 2A**). Antibody responses were even stronger in HIV patients (fold increase 4.99 (4.22; 5.91, p<0.001), their post-immunization HAI titers reaching 2-fold higher levels than those of HCs (p<0.001), and a remarkable 98.4% (CI95% 95.4-99.7) seroprotection rate. One dose of seasonal 2010/2011 vaccine also increased the HAI titers of SOT recipients (fold increase 2.83 (1.64; 3.19), p<0.001). However, HAI-titers and seroprotection rates remained lower than in controls (**Table 2A**). Despite adjustment for age, HAI-titers also remained 65.7% lower in SOT than in HIV patients (p<0.001) similarly primed in 2009 with 2 doses of Pandemrix®, reflecting their lower immune competence. Seroconversion rates, less informative when baseline antibodies are high, were significantly higher in HIV patients (**Table 2A**) despite similar pre-2010 HAI-GMTs, reflecting superior memory responses.

Increasing age significantly impacted the post-2010 antibody responses: each additional 10 years independently reduced post-immunization titers by 20% (HC), 22% (HIV) or 29% (SOT) (**Table 4**). Antibody responses appeared paradoxically better in HIV patients with more advanced disease (**Table 3**), but multivariate analyses identified HAART as independently associated with higher vaccine responses (**Table 4**). Memory responses were lower in MMF-treated patients (-42%), without reaching statistical significance.

The strongest determinant of the 2010/2011 antibody responses was the residual (pre-2010) antibody titer. This influence was positive: post-2010 titers were significantly higher (p<0.001) when persisting antibodies were above as compared to below the group median (**Table 3**). Adding persisting antibodies in the regression analyses indicated that they increased post-2010/2011 titers by 97% (HC), 69.6% (HIV) and 137.6% (SOT) (not shown). As post-2010/2011 HAI-GMT were significantly higher in HIV patients compared to HCs after controlling for age, gender and pre-2010 titers (p<0.001), we focused subsequent analyses on participants followed during 2 seasons.

### Influence of 2009/2010 Immunization on 2010/2011 Memory Responses to Influenza A/09/H1N1

HAI titers elicited in 2009/2010 (post-2009), persisting in 2010/2011 (pre-2010) and reactivated in 2010/2011 (post-2010) were compared for the 191 patients of the 2009/2010/2011 cohort (HC: 69, HIV: 71, SOT: 51) (**Table 2B** **and** **Figure 1E**).

As described [20], [23], post 2009/2010 HAI-GMTs were similar in HIV individuals and HCs, and lower in SOT recipients (**Table 2B** **and** **Figure 1E**). HAI-GMTs declined during the following year and increased after the 2010/2011 immunization. In HCs, post-2010/2011 HAI titers remained significantly lower than in 2009/2010 (240.2 vs 313.9, p<0.001) (**Table 2B** **and** **Figure 1E**). In contrast, higher HAI-GMTs were observed in HIV patients following boosting with 1 dose of seasonal 2010/2011 vaccine as compared to priming with 2 doses of Pandemrix® (HAI: 435.7 vs 338.0, p = 0.03; post-2010/post-2009 GMT ratio: 1.365 (1.034; 1.803), p = 0.03). A similar trend towards higher post-2010/2011 responses was observed in SOT recipients (HAI: 136.0 vs 90.3, p = 0.20; GMT ratio 1.502 (0.992; 2.275), p = 0.055). These better 2010/2011 than 2009/2010 responses were not ascribed to higher immune competence: changes in the medical conditions (7 patients) or antiretroviral therapy (13 changes, including 8 changes of drug class) of HIV patients were not considered as increasing immune competence, and immunosuppression intensity had been reduced in 8 but increased in 8 others SOT recipients.

### Similar 2010/2011 Antibody Responses to Influenza A/09/H3N2 in HIV-infected and Healthy Subjects

To compare the immune competence of HIV and HCs during the 2010/2011 season, we assessed primary antibody responses to the influenza A/09/H3N2 strain, first included in the 2010–2011 seasonal trivalent vaccine. H3N2 HAI-GMTs and seroprotection rates were low prior to immunization and increased following immunization (**Table 2C** **and** **Figure 1F**). H3N2 HAI-GMTs remained relatively low and responses were not significantly different among patients and controls (**Table 2C** **and** **Figure 1F**). Thus, true anamnestic responses characterized by stronger booster 2010/2011 than primary 2009/2010 responses against influenza A/09/H1N1 were observed in well-controlled HAART-treated HIV patients and moderately immunosuppressed SOT recipients, but not in healthy individuals.

## Discussion

How immune competence affects memory responses and whether these may be enhanced by adjuvanted vaccines is not well known. Here we show that antibodies elicited by the AS03-adjuvanted pandemic vaccine (Pandemrix®^)^ persisted at similarly high titers in HIV patients as in HCs up to 12 months after priming. Unexpectedly, memory responses elicited against A/09/H1N1 by non-adjuvanted trivalent 2010/2011 influenza vaccines were stronger in HIV patients primed in 2009 with 2 doses of Pandemrix® than in HCs primed with a single dose. Persisting antibody titers and memory responses were also high – although significantly lower - in SOT recipients.

The duration of vaccine-induced antibodies depends upon peak antibody production by short-lived plasma cells and plasma cell persistence. The rapid disappearance of vaccine antibodies usually observed in HIV patients, as following immunization with non-adjuvanted pandemic vaccines [28], may thus result from low peak responses and/or short-lasting antibody production. In contrast to the conclusion of Cagigi et al [24] that “even if immunisation induces a good antibody response, patients with HIV will not sustain antibody production for as long as healthy individuals because of intrinsic defects in their B cell compartment”, HAI antibodies persisted at similarly high levels in HIV patients and HCs and remained above seroprotective levels in most SOT recipients. We cannot formally demonstrate that this resulted from priming with Pandemrix®, as non-adjuvanted pandemic vaccines were unavailable in Switzerland. However, antibody persistence was better following immunization with AS03-adjuvanted than non-adjuvanted H5N1 vaccines (seroprotection rate 36% vs 6%) [30]. As younger age, antiretroviral therapy and higher CD4 counts favoured antibody persistence, shorter/weaker responses might have been observed in non-HAART treated patients.

Controls reached lower titers following boosting in 2010/2011 than priming in 2009/2010, reflecting the recruitment/activation of fewer antigen-specific B cells by 15 ug of non-adjuvanted than 3.8 ug of AS03-adjuvanted H1N1 antigens. These weaker 2010/2011 responses did not result from an inhibitory influence of higher baseline antibodies, which were positively correlated with booster responses. They are unlikely to reflect altered immune competence, an exclusion recruitment criterion. As non-adjuvanted influenza vaccines essentially reactivate pre-existing memory B cells, it suggests that in HCs 1 dose of Pandemrix® drove more B cells towards antibody-secreting plasma cells than memory B cells.

It is thus quite remarkable that HIV and SOT patients responded with higher titers to the non-adjuvanted 2010/2011 seasonal vaccine as to Pandemrix® in 2009/2010. This is unlikely to reflect higher immune competence: the CD4 counts of HIV patients were not higher in 2010/2011 and the immunosuppression of SOT patients was essentially unchanged. Furthermore, immune competence would not be higher in HIV/SOT patients than in controls, in whom 2010/2011 responses remained lower. As primary 2010/2011 responses to influenza A/09/H3N2 were lower and not different among patients and controls, our observations indicate a true enhancement of responses to the A/09/H1N1 strain. Why did patients raise better memory responses than healthy subjects? One key difference is that patients were primed in 2009 with 2 doses and HC with 1 dose of Pandemrix®. This second primary dose implies an additional exposure to A/09/H1N1 and to the AS03 adjuvant. Whether 2 doses of non-adjuvanted H1N1 vaccine would increase subsequent memory responses may not be excluded. But memory B-cells were indeed reported as more numerous in HIV patients immunized with Pandemrix® than non-adjuvanted A/09/H1N1 vaccines [31]. Our field study was not planned to compare the influence of one versus two doses of Pandemrix® on memory responses, which would not have been allowed by the Swiss authorities. Nevertheless, the most likely explanation to our observations is that 2 doses of AS03-adjuvanted vaccines provided a more efficient induction of memory responses than a single vaccine dose. This was sufficient to overcome the potential deficiencies of the B cell compartment caused by HIV infection. It even proved beneficial in SOT patients, although their antibodies persisted/were reactivated at lower levels than in HIV patients similarly primed with 2 doses of adjuvanted pandemic vaccine, indicating lower immune competence. That adjuvanted vaccines may improve antibody persistence and memory responses of immunocompromised patients is worth studying further.

## Supporting Information

Figure S1 **Study Flow Chart.**
(TIF)

Table S1 **Description of Adverse Reactions.**
(DOCX)

Protocol S1 **Trial Protocol.**
(PDF)
