# Supplementary figures and images for: Higher Memory Responses in HIV-Infected and Kidney Transplanted Patients than in Healthy Subjects following Priming with the Pandemic Vaccine
Source: PLoS One. 2012 Jul 27;7(7):e40428. doi: 10.1371/journal.pone.0040428 (PMC3407205; doi:10.1371/journal.pone.0040428)

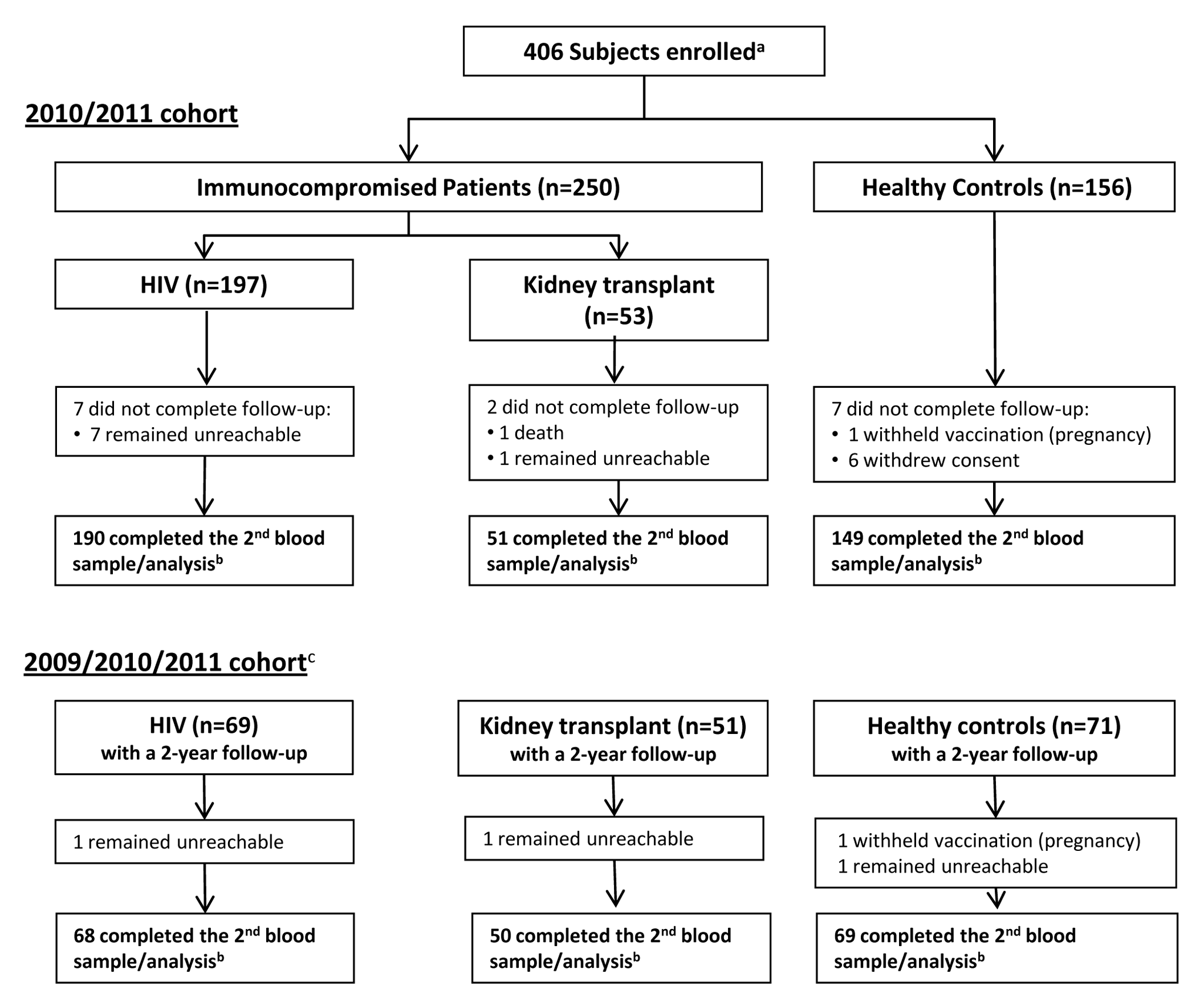

Supplement: Figure S1 — Study Flow Chart. (TIF) [file pone.0040428.s001.tif]
