# Supplementary material for: Higher Memory Responses in HIV-Infected and Kidney Transplanted Patients than in Healthy Subjects following Priming with the Pandemic Vaccine
Source: PLoS One. 2012 Jul 27;7(7):e40428. doi: 10.1371/journal.pone.0040428 (PMC3407205; doi:10.1371/journal.pone.0040428)
Supplement: Table S1 — Description of Adverse Reactions. (DOCX) [file pone.0040428.s002.docx]

| **Supplementary Table 1. Adverse reactions** | | | |  |  |  |  |  |  |  |
| --- | --- | --- | --- | --- | --- | --- | --- | --- | --- | --- |
|  |  |  |  |  |  |  |  |  |  |  |
| **Adverse Reactions, Number of patients, N (%) (95%CI)** | | **Controls** | | **HIV** | | **Tranplants (kidney)** | | **Tranplants (lung)** | |  |
|  |  | **N** | **% with AE** | **N** | **% of AE** | **N** | **% with AE** | **N** | **% with AE** | **p-value *** |
| *Any reaction* | | 110/149 | 73.8 (66.0;80.7) | 102/190 | 53.7 (46.3;60.9) | 32/51 | 62.7 (48.1;75.9) | 9/9 | 100.0 (66.4;100.0) | <0.001 |
| *Any pain* | | 89/149 | 59.7 ( 51.4 ; 67.7 ) | 80/190 | 42.1 ( 35 ; 49.5 ) | 18/51 | 35.3 ( 22.4 ; 49.9 ) | 5/9 | 55.6 ( 21.2 ; 86.3 ) | <0.001 |
| *Pain*** | *None* | 60/149 | 40.3 ( 32.3 ; 48.6 ) | 110/189 | 58.2 ( 50.8 ; 65.3 ) | 33/51 | 64.7 ( 50.1 ; 77.6 ) | 4/9 | 44.4 ( 13.7 ; 78.8 ) | 0.01 |
|  | *Mild* | 69/149 | 46.3 ( 38.1 ; 54.7 ) | 55/189 | 29.1 ( 22.7 ; 36.1 ) | 14/51 | 27.5 ( 15.9 ; 41.7 ) | 4/9 | 44.4 ( 13.7 ; 78.8 ) |  |
|  | *Moderate* | 17/149 | 11.4 ( 6.8 ; 17.6 ) | 20/189 | 10.6 ( 6.6 ; 15.9 ) | 3/51 | 5.9 ( 1.2 ; 16.2 ) | 1/9 | 11.1 ( 0.3 ; 48.2 ) |  |
|  | *Severe* | 3/149 | 2 ( 0.4 ; 5.8 ) | 4/189 | 2.1 ( 0.6 ; 5.3 ) | 1/51 | 2.0 ( 0.0 ; 10.4 ) | 0/9 | 0.0 ( 0.0 ; 33.6 ) |  |
| *Any redness* | | 22/149 | 14.8 ( 9.5 ; 21.5 ) | 13/190 | 6.8 ( 3.7 ; 11.4 ) | 5/51 | 9.8 ( 3.3 ; 21.4 ) | 4/9 | 44.4 ( 13.7 ; 78.8 ) | 0.058 |
| *Redness* | *0 cm* | 127/149 | 85.2 ( 78.5 ; 90.5 ) | 177/189 | 93.7 ( 89.2 ; 96.7 ) | 46/51 | 90.2 ( 78.6 ; 96.7 ) | 5/9 | 55.6 ( 21.2 ; 86.3 ) | <0.001 |
|  | *1-3 cm* | 19/149 | 12.8 ( 7.9 ; 19.2 ) | 8/189 | 4.2 ( 1.8 ; 8.2 ) | 4/51 | 7.8 ( 2.2 ; 18.9 ) | 3/9 | 33.3 ( 7.5 ; 70.1 ) |  |
|  | *>3 cm* | 3/149 | 2.0 ( 0.4 ; 5.8 ) | 4/189 | 2.1 ( 0.6 ; 5.3 ) | 1/51 | 2.0 ( 0.0 ; 10.4 ) | 1/9 | 11.1 ( 0.3 ; 48.2 ) |  |
| *Any swelling* | | 25/149 | 16.8 ( 11.2 ; 23.8 ) | 16/190 | 8.4 ( 4.9 ; 13.3 ) | 5/51 | 9.8 ( 3.3 ; 21.4 ) | 3/9 | 33.3 ( 7.5 ; 70.1 ) | 0.054 |
| *Swelling* | *0cm* | 124/149 | 83.2 ( 76.2 ; 88.8 ) | 174/189 | 92.1 ( 87.2 ; 95.5 ) | 46/51 | 90.2 ( 78.6 ; 96.7 ) | 6/9 | 66.7 ( 29.9 ; 92.5 ) | 0.049 |
|  | *1-3cm* | 21/149 | 14.1 ( 8.9 ; 20.7 ) | 9/189 | 4.8 ( 2.2 ; 8.8 ) | 4/51 | 7.8 ( 2.2 ; 18.9 ) | 2/9 | 22.2 ( 2.8 ; 60.0 ) |  |
|  | *>3cm* | 4/149 | 2.7 ( 0.7 ; 6.7 ) | 6/189 | 3.2 ( 1.2 ; 6.8 ) | 1/51 | 2.0 ( 0.0 ; 10.4 ) | 1/9 | 11.1 ( 0.3 ; 48.2 ) |  |
| *Fever* | *None* | 145/149 | 97.3 (93.3;99.3) | 185/190 | 97.4 (94.0;99.1) | 51/51 | 100.0 (93.0;100.0) | 9/9 | 100.0 (66.3;100.0) | 0.8 |
|  | ≥ *38° C* | 4/149 | 2.7 (0.7;6.7) | 5/190 | 2.6 (0.9;6.0) | 0/51 | 0.0 (0.0;7.0) | 0/9 | 0.0 ( 0.0 ; 33.6 ) |  |
|  | *≥ 38.5° C* | 1/148 | 0.7 (0.0;3.7) | 3/190 | 1.6 (0.3;4.5) | 0/51 | 0.0 (0.0;7.0) | 0/9 | 0.0 ( 0.0 ; 33.6 ) |  |
|  | *≥ 39° C* | 0/148 | 0.0 (0.0;2.5) | 2/190 | 1.1 (0.1;3.8) | 0/51 | 0.0 (0.0;7.0) | 0/9 | 0.0 ( 0.0 ; 33.6 ) |  |
| *Chills* |  | 4/149 | 2.7 ( 0.7 ; 6.7 ) | 12/190 | 6.3 ( 3.3 ; 10.8 ) | 2/51 | 3.9 ( 0.5 ; 13.5 ) | 0/9 | 0.0 ( 0.0 ; 33.6 ) | 0.29 |
| *Sudation* | | 5/149 | 3.4 ( 1.1 ; 7.7 ) | 10/190 | 5.3 ( 2.6 ; 9.5 ) | 2/51 | 3.9 ( 0.5 ; 13.5 ) | 2/9 | 22.2 ( 2.8 ; 60.0 ) | 0.74 |
| *Myalgia* |  | 18/149 | 12.1 ( 7.3 ; 18.4 ) | 34/190 | 17.9 ( 12.7 ; 24.1 ) | 6/51 | 11.8 ( 4.4 ; 23.9 ) | 2/9 | 22.2 ( 2.8 ; 60.0 ) | 0.26 |
| *Arthralgia* | | 5/149 | 3.4 ( 1.1 ; 7.7 ) | 19/190 | 10.0 ( 6.1 ; 15.2 ) | 4/51 | 7.8 ( 2.2 ; 18.9 ) | 0/9 | 0.0 ( 0.0 ; 33.6 ) | 0.0499 |
| *Fatigue* |  | 24/149 | 16.1 ( 10.6 ; 23 ) | 38/190 | 20.0 ( 14.6 ; 26.4 ) | 15/51 | 29.4 ( 17.5 ; 43.8 ) | 4/9 | 44.4 ( 13.7 ; 78.8 ) | 0.12 |
| *Anorexia* | | 1/149 | 0.7 ( 0.0 ; 3.7 ) | 12/190 | 6.3 ( 3.3 ; 10.8 ) | 2/51 | 3.9 ( 0.5 ; 13.5 ) | 2/9 | 22.2 ( 2.8 ; 60.0 ) | 0.02 |
| *Headaches* | | 18/149 | 12.1 ( 7.3 ; 18.4 ) | 24/190 | 12.6 ( 8.3 ; 18.2 ) | 12/51 | 23.5 ( 12.8 ; 37.5 ) | 2/9 | 22.2 ( 2.8 ; 60.0 ) | 0.1 |
| *Other* |  | 23/149 | 15.4 ( 10.0 ; 22.3 ) | 23/190 | 12.1 ( 7.8 ; 17.6 ) | 14/51 | 27.5 ( 15.9 ; 41.7 ) | 0/9 | 0.0 ( 0.0 ; 33.6 ) | 0.03 |
| * Between controls, HIV-infected patients and kidney transplant recipients | | | | | | | |  |  |  |
| 95%CI : 95% confidence interval | | |  |  |  |  |  |  |  |  |
| **Mild: no interference with activities; Moderate: interference with normal activities; | | | | | | | |  |  |  |
| Severe: prevented daily activity or required medical attention | | | | | |  |  |  |  |  |
