# Supplementary material for: Higher Memory Responses in HIV-Infected and Kidney Transplanted Patients than in Healthy Subjects following Priming with the Pandemic Vaccine
Source: PLoS One. 2012 Jul 27;7(7):e40428. doi: 10.1371/journal.pone.0040428 (PMC3407205; doi:10.1371/journal.pone.0040428)
Supplement: Protocol S1 — Trial Protocol. (PDF) [file pone.0040428.s003.pdf]

Study protocol

Prospective cohort study of the influence of age, underlying disease and immunosuppression on vaccine responses to influenza A H1N1/09 immunization in high-risk patients

Running title: Influence of age, underlying diseases and immunosuppression on vaccine responses to influenza A H1N1/09 immunization

Sponsor and Principal investigator

Prof. Claire-Anne Siegrist
Centre de Vaccinologie et d'Immunologie Néonatale, Faculté de Médecine,
Université Genève (UNIGE)
Unité d'immuno-vaccinologie, Département de l'Enfant et de l'Adolescent,
Hôpitaux universitaires de Genève (HUG)

Co-Investigators

Prof. Laurent Kaiser, Dr Yves Thomas, Dr Werner Wunderli
Laboratoire de Virologie, Département de médecine interne, HUG

Prof. Jules Desmeules, Dr Christophe Combescure
Centre de Recherche Clinique, HUG et Faculté de Médecine, UNIGE

Dr Klara Posfay-Barbe
Plateforme de recherche clinique pédiatrique, Département de l'Enfant et de l'Adolescent,
HUG et Faculté de Médecine, UNIGE

Prof. Cem Gabay
Service de Rhumatologie, Département de médecine interne, HUG

Prof. Pierre-Yves Dietrich, Dr Eddy Roosnek, Prof. Jacob Passweg
Service d'oncologie et Service d'hématologie, Département de médecine interne, HUG

Dr Alexandra Calmy, Dr Christian van Delden, Prof. Bernard Hirschel
Service des maladies infectieuses, Département de médecine interne, HUG

Dr Christian van Delden
Service de transplantation, Département de chirurgie, HUG

Prof. Joerg Seebach, Dr Camillo Ribi
Service d'Immunologie, Département de médecine interne, HUG

Signature form

I, the undersigned, have reviewed this Protocol, including Appendices. I will conduct the clinical study as described and I will adhere to GCP/ICH and all the ethical and regulatory considerations stated (OClin and LPTh).

Claire-Anne Siegrist	CaSiegrist	20.7.2010
**Principal investigator**	**Signature**	**Date**

Prospective cohort study of the influence of age, underlying disease and immunosuppression on vaccine responses to influenza A H1N1/09 immunization in high-risk patients
Title

Centre de vaccinologie et d'immunologie néonatale
Centre Médical Universitaire, 1 rue Michel-Servet, 1211 Genève 4
Institution and address

022 379 57 78, 079 213 92 90
Phone

Table of contents

Synopsis	4
Study flow chart	5
1. Justification for the proposed study	6
2. Objectives: main questions to be answered through this research project	9
3. Study design	10
4. Study population	12
5. Study treatment	19
6. Evaluation criteria	19
7. Data collection and management	21
8. Adverse events management	22
9. Quality control and assurance	23
10. Statistics	24
11. Ethical considerations	29
12. Publication policy	30
13. Financing and insurance	30
14. 2010 study extension	31
15. Annexes	33

Synopsis

Study title	Prospective cohort study of the influence of age, underlying diseases and immunosuppression on vaccine responses to influenza A H1N1/09 immunization in high-risk patients
Running title (French)	Etude des réponses au vaccin contre la grippe A H1N1/09, dite « porcine »
Investigators	Principal Investigator: Prof. Claire-Anne Siegrist<br>Co-Investigators : Prof. Laurent Kaiser, Dr Yves Thomas, Prof. Werner Wunderli, Dr Klara Posfay-Barbe, Prof. Jules Desmeules, Dr Christophe Combescure, Prof. Cem Gabay, Prof. Pierre-Yves Dietrich, Prof. Jacob Passweg, Dr Eddy Roosnek, Dr Alexandra Calmy, Prof. Bernard Hirschel, Dr Christian van Delden, Dr Camillo Ribi, Prof. Joerg Seebach
Study centre	Hôpitaux Universitaires de Genève
Study period	October 2009 – September 2011
Objectives	To assess the influence of age, underlying disease or immunosuppressive conditions on vaccine responses to influenza A H1N1/09 immunization
Methodology	Prospective, open-label, parallel-cohorts study
Number of participants	1250 patients and 250 controls (maximum)
Inclusion criteria:	Patients at high risks of influenza A H1N1/09 complications (respectively their control contacts) to whom immunization has been recommended according to the official recommendations (EKIF/BAG) and usual routine guidelines and accepted by the patient/parents, followed in Geneva during the 2009-2010 influenza season and who provided a signed informed consent.
Non-inclusion criteria:	Cases: Failure or refusal to provide sufficient blood specimens for the determination of vaccine antibody responses; Controls: underlying diseases or any immunosuppressive condition.
Evaluation	Specific antibody responses (primary endpoints: inhibition of hemagglutination); neutralizing antibodies and T cell responses (secondary endpoints); solicited vaccine adverse events (descriptive); markers of underlying disease activity / graft function.
Duration of study	(participants): from 1st vaccine dose to 4-6 weeks after the last vaccine dose.
Statistical Methods	Immunogenicity endpoints and confidence bounds will be calculated for each cohort, group and sub-group and compared (by Chi-square or Fisher exact test, and Wilcoxon’s test or Kruskal-Wallis test) within a group, a cohort and across cohorts. The influence of variables will be tested in an univariate way. A logistic model will be constructed to study the factors likely to explain the proportion reaching the primary endpoints. A multivariate analysis (logistic regression) will compare patients and healthy controls, while adjusting for other variables. The goodness-of-fit will be analysed using the Hosmer-

Lemeshow' test. Recorded solicited adverse events will be described and compared using non-parametric methods. All tests will be two-sided.

Study flow chart

	Visit 1	Visit 2	
	≤ 24h of 1st vaccine	≥ 4 wks to ≤ 6 wks of 1st vaccine	of 2nd vaccine
Informed consent	X		
Data collection (diseases and treatment)	X		
Blood sample			
Controls[1]	X	X	
Patients[2]	X	(X)	X
Data collection (vaccine adverse events)			
Controls[1]		X	
Patients[2]			X

[1] For control patients, to whom only 1 vaccine dose is now recommended, the 2nd visit will take place 4-6 weeks after the first immunization.

[2] To allow optimal comparisons with control patients, who will be recruited among the contacts of patients with cancer or rheumatologic diseases, we will ask patients from the cancer and rheumatologic disease cohorts whether they agree to provide an additional blood sample to measure there responses to the first vaccine dose. This additional venous bleed will be optional.

1. Justification for the proposed study

a. Influenza A H1N1 disease

Influenza A H1N1 is a new virus that emerged in spring 2009 and rapidly spread around the world, causing a pandemic. Given the lack of herd immunity and the transmissibility of this new pandemic influenza A H1N1 2009 it is expected that a large proportion of the population, including those with underlying medical conditions will be exposed in the community. The evaluation of the morbidity / lethality caused by influenza A H1N1/09 is still ongoing. Nevertheless, it is recognized that the transmissibility and risk of this new influenza infection is high. The main complications of influenza are exacerbations of previous lung diseases or asthma as well as bacterial complications. Exacerbations of lung disease lead to frequent medical interventions, antibiotic overuse and hospitalizations. In addition influenza is known to cause viral pneumonia (a condition often unrecognized), which may be further complicated by bacterial pneumonia and/or Acute Respiratory Distress Syndrom (ARDS) and death. Other severe complications have been reported, among which myocarditis and central nervous events (seizures, encephalitis) are the most feared. In the US, UK, and Australia these complications required the hospitalization of up to 13% of cases with a positive A H1N1/09 test, lead to ICU admission in approximately 20% of them, and caused the death of 1-10 of 10'000 such patients. These complications are fortunately rare in healthy individuals, but not so rare in patients with chronic diseases - as approximately 60% hospitalizations and most deaths occur in subjects vulnerable because of underlying risk factors.

The identified risk factors for influenza A H1N1/09 are similar to those that increase the risks of complications from seasonal influenza : young age (< 12 months, prematurity) and chronic diseases affecting the heart, the lungs or the immune system as well as pregnancy.

The WHO, the European Center for Disease Control and national health authorities world wide have thus recommended the priority immunization of high-risks patients. In Switzerland, the Commission fédérale pour les vaccinations recommends the priority immunization of pregnant women and of children from the age of 6 months, adolescents and adults with underlying diseases affecting their heart (cardiac malformation, pulmonary hypertension, cardiac insufficiency, etc.…), their lungs (asthma, cystic fibrosis, chronic pulmonary diseases, neurological or muscular disorders, etc.) or their immune system (prematurity, immunosuppressive treatment, cancer, HIV-1 infection, congenital immunodeficiencies, renal insufficiency, hemoglobinopathies, chronic metabolic disorders and any chronic disease reducing immune competence) (www.ofsp.ch, accessed on August 25th – document dated 13th August 2009). This recommendation will be officially endorsed / published by the Federal Office of Public Health on September 28, 2009 *(date of publication in the BAG Bulletin).*

b. Influenza A H1N1/09 vaccines

To face the threat of this new virus, seasonal influenza vaccines have been adapted to include the novel influenza A H1N1/09 strain. The Federal Office of Public Health has signed contracts with 2

vaccine producers (Glaxo SmithKline, GSK, and Novartis Vaccines) which will provide vaccines for the swiss population. The influenza A H1N1/09 vaccines produced by GSK and Novartis are similar: they include influenza A H1N1/09 purified antigens formulated in a lipid-based adjuvant (squalene) which forms an emulsion and improves antigen presentation to the immune system. These 2 vaccines have been evaluated by the European Medicines Agency and Swissmedic, and have been licensed by Swissmedic. According to the product homologation / official recommendations, healthy adults at high-risk of transmission may be immunized with a single dose of Pandemrix® (GSK), whereas 2-doses at a ≥ 3 weeks interval are recommended for immunocompromised patients. Focetria® (Novartis) should be used for children (2 doses) and pregnant women (1 dose).

Influenza vaccine reactogenicity and safety are essentially driven by the adjuvant – rather than by the specific antigens included in a vaccine. Thus, H1N1/09 vaccines will be licensed based also on the evidence collected 1) during the development of an H5N1 avian pandemic vaccine and 2) during the development and clinical use of the Novartis adjuvanted seasonal influenza vaccine (Fluad®). Fluad® has been distributed to more than 100'000 individuals (from newborns to seniors) during clinical studies and to 47 million individuals worldwide, which results into a large safety data base. Importantly, these vaccines do not contain any live virus, such that they may be administered even to immunodeficient patients.

In contrast, these vaccines will be licensed with much more limited information regarding their immunogenicity / efficacy – as this is dependent upon the selected viral antigens. It is expected that 2 vaccine doses (at a minimal interval of 3 weeks) should result into a large proportion of healthy adults raising protective seroresponses – although the amplitude of this response and the exact proportion of those that will achieve protective titers is yet unknown. The Commission fédérale pour les vaccinations (CFV/EKIF) and the Federal Office of Public Health recommend that high-risk patients (with underlying cardiovascular or pulmonary disease or limited immune competence) be immunized as soon as the vaccines will have been licensed by Swissmedic (Bulletin OFSP, 20.10.2009).

c. Vaccine responses in patients at high risks of influenza complications

Protective vaccine responses will not be elicited by adjuvanted influenza vaccines in 100% healthy individuals. Patients at high risks of complications from influenza A H1N1/09 may have a well preserved, limited or significantly depressed immune response capacity: although specific data is not yet available, patients with well-compensated cardiac or pulmonary diseases are expected to raise similar vaccine responses as would healthy patients, patients with chronic organ diseases that affect their state of health (such as renal insufficiency) could respond less well, whereas immunocompromised patients may succeed or fail to raise vaccine responses depending upon their individual level of immune competence.

Consequently, it is not possible to predict that 2 vaccine doses will generate sufficient protection for additional prophylactic / therapeutic measures to be alleviated. This is of major clinical relevance for

high risk group patients given the burden of prophylactic/therapeutic measures they require. Indeed, in the absence of the demonstration of positive vaccine responses, avoiding exposure as much as possible and empiric treatment with oseltamivir will remain required at each febrile influenza-like illness during many months – as influenza pandemic usually occur in several waves. Although correlates of protection have not yet been exactly defined for influenza A H1N1/09, antibody responses (assessed through inhibition of hemagglutination and/or microneutralisation) are known to largely mediate this protection and constitute the basis for the licensing of influenza vaccines.

In addition, this prospective collection of immune responses will provide original and most needed observations in the field. The ability of distinct patients populations with distinct degree and type of immunosuppressive conditions to raise vaccine responses to a specific common antigen have never been studied in such a systematic and standardized manner. The fact the targeted agent has not circulated in the population in the previous years is also an unique opportunity to assess essentially primary vaccine responses.

d. Safety of adjuvanted influenza A(H1N1) vaccines (admendment)

The safety of adjuvanted influenza A(H1N1) vaccines has been questioned by the media at large. Fears that adjuvanted vaccines may have non-specific deleterious influences on the immune system and thus on underlying diseases have led some patients and physicians not to accept / recommend the use of adjuvanted vaccines to high-risk patients. After approximately 1’000 immunizations of study participants, our impression is that these vaccines are safe – which is also indirectly supported by the world-wide pharmacovigilance reports.

To more objectively demonstrate this vaccine safety in high-risk patients, we will include the evaluation of markers of underlying disease activity and/or graft function. Analyses will be performed at the final blood draw, and compared to baseline values retrieved from the medical chart (most recent control) or performed on serum harvested at Day 0.

2. Objectives: main questions to be answered through this research project

The general objective of this parallel-cohort study is to provide information on determinants that affect vaccine immune responses

1) within a given cohort (e.g. influence of CD4 T cell count among HIV-1 patients, of immunosuppressive regimen among patients with rheumatologic disorders, of time elapsed after liver transplant, etc).
2) as compared to healthy individuals
3) across the various cohorts – allowing to progress towards the identification of makers (or surrogates) of immune competence.

Towards this general aim, primary and secondary objectives have been identified.

Primary objectives : antibody responses

- Which proportion of high-risks patients raise antibody responses associated with protection following one or two doses of influenza A H1N1/09 vaccines, as compared to healthy individuals after 1 dose ? What is the magnitude (titer) of their antibody response?
- How is the proportion of seroresponders and the magnitude of the response influenced by age / underlying diseases / type of immunosuppression / number of vaccine doses? What is their influence on the magnitude (titer) of antibody responses?

Secondary objectives: T cell responses

- In which proportion of high-risks patients does influenza A H1N1/09 immunization induce a specific CD4 lymphocyte T cells response following 2 vaccine doses? How is this proportion and the magnitude of cellular responses affected by age / underlying diseases / type of immunosuppression? Does age / underlying diseases / immunosuppression affect the type of cytokines produced by vaccine-induced T cells?
- What is the correlation between antibody and cellular responses? How does age / underlying diseases / type of immunosuppression affect this correlation.

Secondary objectives: vaccine safety

A vaccine safety objective has been added given the need to address vaccine fears by objectively demonstrating the safety of adjuvanted vaccines. This will be assessed by the proportion of patients with a deterioration of their medical condition, as reflected by increased requirements for immunosuppression, increased markers of underlying disease activity, or reduced graft function – as appropriate.

3. Study design

3.1 Overall description

This prospective, open-labeled, parallel-cohort study will enroll subjects to whom influenza A H1N1/09 immunization will have been recommended according to the official recommendations from the EKIF/BAG and the usual guidelines – because of their high risks of complications (patients) or of transmission (household contacts, health professionals). This study will only recruit individuals who have previously accepted to be immunized - no subject will be immunized for the sake of this study. The study will include 1) the collection of clinical information to characterize the underlying disease / treatment and potential vaccine-associated solicited adverse events, 2) two venipunctures, at time of the first vaccine dose and 4 to 6 weeks after the last dose, to allow the assessment of vaccine responses.

3.2 Primary and secondary endpoints

Primary endpoints: Three co-primary immunogenicity endpoints after vaccination were chosen according to international guidelines used to evaluate influenza vaccines : 1) the proportion of subjects with serum hemaggglutination inhibition (HAI) antibody ≥ 1:40, 2) the proportion of subjects with either seroconversion or a significant increase in antibody titer, and 3) the factor increase in the geometric mean titer after 2 vaccine doses.

Secondary endpoints: The secondary immunogenicity endpoints will include 1) the same endpoints applied to the quantification of neutralizing antibody titers, 2) the proportion of subjects with detectable influenza A H1N1/09 specific CD4+ T cell responses after 2 vaccine doses, 3) the proportion of patients with a deterioration of their medical condition, as reflected by increased requirements for immunosuppression, increased markers of underlying disease activity, or reduced graft function – as appropriate.

Note : Assessing vaccine-associated adverse events in patients at high risks of influenza A H1N1/09 is not a primary objective of this study. However, according to good-clinical-practice guidelines we will monitor the frequency, duration and intensity of solicited adverse events during the 7 days after each vaccination and the incidence of serious adverse events until 4 weeks after the 2nd vaccine dose. This will allow establishing potential correlations between inflammatory and antibody / cellular responses.

3.3 Randomisation and blinding

There will be no randomization or observer-blinding in this parallel-cohorts open-label study. However, all laboratory analyses will be performed by technical collaborators unaware of the patients' clinical conditions or treatment.

3.4 Study duration for subjects

Study duration for participants will be the time between the two study visits, i.e. between within 24h of the first immunization and the post-immunization visit scheduled 4-6 weeks after the last vaccine dose. Immunization will include 2 doses at least 3 weeks apart – and 1 dose in healthy adult contacts of high-risk patients. Thus, study duration will be approximately 2 months and will not exceed 4 months.

3.5 Data collected in the CRF

Data collected in the CRF will include a list of demographic variables common to each cohort, as well as cohort-specific variables related to the underlying disease and treatment (cf Annex 3).

Information on the frequency, duration and intensity of solicited adverse events during the 7 days after each vaccine dose, and the incidence of serious adverse events until 4 weeks after the 2nd vaccine dose, will be collected as well (cf Annex 3).

Withdrawal procedure:
Study participants will be free to withdraw from the study at any time, without having to provide any explanation. Routine care will not be affected by the study withdrawal, and pandemic H1N1/2009 will also be offered to patients not included into the present study.

4. Study population

This parallel-cohort study will include several cohorts – each led by a main investigator and his/her team of collaborators.

Remark: As vaccination is also recommended for pregnant women, women who correspond to the inclusion criteria of one of the cohort (HIV infection for example), will not be excluded from the study. The vaccine used would then be Focetria® (as explained page 7).

4.1 HIV-infected patients (N max = 200)

Main investigator : Dr Alexandra Calmy

Collaborators : Prof. Bernard Hirschel, Dr Sarra Inoubli, Alain Nguyen, Prof. Claire-Anne Siegrist, Prof. Laurent Kaiser, Dr Klara Posfay-Barbe

The cohort of HIV-1 infected patients enrolled in the Swiss HIV Cohort Study and followed in the HUG includes 600 patients, including 346 patients with a CD4 T cell count greater than 500/ml (group 1) and 253 patients with a CD4 T cell count lower than 350/ml (group 2). 93% are under antiretroviral therapy (HAART) treatment and their mean age is 45 years. The selection of these 2 patient groups will ensure the inclusion of patients with a significantly better preserved or a more limited pool of CD4 T cells. International guidelines recommend immunizing all HIV-infected patients regardless of CD4 cell count or HIVRNA against influenza and pneumococcal infections. An immunological response to seasonal inactivated influenza vaccine in HIV infected patients is satisfactory when CD4 cells is above 200 cells/mm3; in one study, HIVRNA was an important variable of the immune response in these patients.

Inclusion criteria :

1. HIV-positive patients under HAART to whom influenza A H1N1/09 has been prescribed by the physician in charge according to the recommendation of the BAG, and who have chosen to be immunized
2. Who have CD4 T cells either > 500 (group 1) or < 350 / mm^3 (group 2)
3. Age ≥ 18 years
4. Followed up in Geneva during the next 3 months
5. Able to sign an informed consent form.

Non inclusion criteria :

1. Refusal or failure to provide sufficient blood at least for the determination of antibody responses.
2. Completion of the enrollment phase of the study (N max = 200, i.e. 100 patients per group).

Sample size

The influence of HIV-1 infection on vaccine-driven responses to influenza A H1N1/09 is unknown, precluding calculation of the sample size needed to ensure statistical power. A maximal sample size of 100 subjects per study group (i.e. 100 patients with CD4+ T cells > 500 and 100 with CD4+ T cells < 350) has been arbitrarily selected based on similar studies. This number could be reduced by the interruption of enrolment, should subsequent available evidence indicate that a smaller sample size is sufficient. Note : all HIV-infected patients will receive 2 vaccine doses, allowing direct comparisons between patients with >500 or <100 CD4+ T cells.

Complementary analyses: the number of viral copies/ml (HIV viremia) will be assessed at the final study visit (4-6 weeks after the last vaccine dose) and compared to baseline values extracted from the medical file (most recent control).

4.2 Patients with inflammatory rheumatic diseases (N max = 300)

Main investigator : Prof. Cem Gabay

Collaborators : Prof. Pierre-André Guerne and other members of the Division of Rheumatology, Prof. Joerg Seebach, Dr Camillo Ribi, Prof. Claire-Anne Siegrist, Prof. Laurent Kaiser, Dr Klara Posfay-Barbe

Patients with inflammatory rheumatic diseases, including rheumatoid arthritis, spondyloarthropathies, connective tissue diseases and systemic necrotizing vasculitis exhibit some form of immunodeficiency related to their diseases and the use of immunosuppressive drugs. Classical immunosuppressive drugs include methotrexate, leflunomide, azathioprine, mycophenolate mofetil, cyclophosphamide, and cyclosporine A. These drugs also termed disease modifying anti-rheumatic drugs (DMARDs) are primarily used in patients with rheumatoid arthritis and other forms of arthritis. These treatments increase the risk of superimposed infections. In addition, the current use of biological agents such as cytokines inhibitors (tumor necrosis factor (TNF) antagonists, interleukin (IL)-6 inhibitors, IL-1 inhibitors), blockers of T cell co-signaling (abatacept); and B-cells depleting therapies (rituximab) have all been shown to be associated with an increased risk of serious complications from infection. Thus, since a few years several Societies of Rheumatology in different Countries have recommended to immunize all patients under biological therapies against influenza and pneumococcal infections.

Patients will be recruited in the following groups (N = maximum number):

1. Patients with rheumatoid arthritis treated with classical DMARDs (N=75)
2. Patients with rheumatoid arthritis treated with TNF inhibitors (N=50)
3. Patients with rheumatoid arthritis treated with B cell depleting therapy (N=35)
4. Patients with spondyloarthropathies treated with classical DMARDs (N=40)
5. Patients with spondyloarthropathies treated with TNF inhibitors (N=50)
6. Patients with systemic lupus erythematosus or necrotizing vasculitis treated with classical DMARDs (N=50)

Inclusion criteria :

1. Patients with rheumatic diseases treated with immunosuppressive drugs/biological agents to whom influenza A H1N1/09 has been prescribed by the physician in charge according to the recommendation of the BAG and who have chosen to be immunized
2. Age ≥ 18 years
3. Followed up in Geneva during the next 3 months
4. Able to sign an informed consent form.

Non-inclusion criteria :

1. Refusal or failure to provide sufficient blood at least for the determination of antibody responses.
2. Completion of the enrollment phase of the study (N max = 300).

Sample size

The influence of underlying disease and immunosuppression on vaccine-driven responses to influenza A H1N1/09 is unknown, precluding calculation of the sample size needed to ensure statistical power. A maximal sample size of 300 subjects has been selected based on the number of patients actively followed. This number could be reduced by the interruption of enrolment, should subsequent available evidence indicate that a smaller sample size is sufficient. Note: all patients will receive 2 vaccine doses, allowing direct comparisons between patients of various treatment groups. We expect that a significant proportion of patients will consent to provide blood after their first vaccine dose, allowing direct comparisons with their healthy contacts.

Complementary analyses: C3, C4, thrombocytes, serum creatinin and anti-dsDNA antibodies (group 6, lupus), CRP, serum creatinin and ANCA antibodies (group 6, vasculitis) and CRP (groups 1-5) will be assessed at the final study visit (4-6 weeks after the last vaccine dose) and compared to baseline values extracted from the medical file (most recent control) or assessed on stored Day 0 samples.

4.3 Patients with hematologic or solid malignancies (N max = 300)

Main investigator : Prof. Pierre-Yves Dietrich

Collaborators : Prof. Jakob Passweg, Dr Assma Ben Aïssa, Dr Alexandre Bodmer, Dr Valérie Dutoit, Dr Laurence Favet, Dr Claudine Helg, Dr Andreas Hottinger, Dr Robert Kridel, Dr Nicolas Mach, Dr Monika Nagy, Dr Eddy Roosnek, Prof. Claire-Anne Siegrist, Prof. Laurent Kaiser, Dr Klara Posfay-Barbe

Patients with hematologic or solid malignancies may suffer from mild, significant or major limitations of their immune competence depending upon the disease condition and treatment regimen. Consequently, influenza immunization is recommended to all patients with cancer. In this study, we will offer a participation to patients suffering from disease-inducing immunosuppression (lymphoma), to those with severe chemotherapy-induced lymphopenia (glioma), to those with neutropenia-inducing

chemotherapies (for pulmonary, head/neck, digestive tract or breast cancer) and to patients after allogenic bone marrow transfer.

Patients will be recruited in the following groups (N = maximum enrollment):

1. Lymphoma (n = 60)
2. Glioma (n = 40)
3. Pulmonary or head/neck cancer (n = 50)
4. Digestive tract carcinoma (n = 50)
5. Breast cancer (n = 50)
6. Patients after stem cell transplantation (n = 50)

Inclusion criteria :

1. Patients with hematologic or solid malignancies to whom influenza A H1N1/09 has been prescribed by the physician in charge according to the recommendation of the BAG and who have chosen to be immunized
2. Age ≥ 18 years
3. Followed up in Geneva during the next 3 months
4. Able to sign an informed consent form.

Non-inclusion criteria :

1. Refusal or failure to provide sufficient blood at least for the determination of antibody responses.
2. Completion of the enrollment phase of the study (cf N max per group).

Sample size

The influence of underlying disease and immunosuppression on vaccine-driven responses to influenza A H1N1/09 is unknown, precluding calculation of the sample size needed to ensure statistical power. A maximal sample size of 300 subjects has been arbitrarily selected based on similar studies and number of patients actively followed. This number could be reduced by the interruption of enrolment, should subsequent available evidence indicate that a smaller sample size is sufficient. Note: all patients will receive 2 vaccine doses, allowing direct comparisons between patients of various disease / treatment groups. We expect that a significant proportion of patients will consent to provide blood after their first vaccine dose, allowing direct comparisons with their healthy contacts.

4.4 Solid organ transplant patients (N max = 250)

Main investigator : Dr Christian van Delden

Collaborators : Dr. Paola Gasche-Soccal, Dr Karine Hadaya, Dr. Emiliano Giostra, Prof Thierry Berney, Dr. Stephane Noble, Prof. Claire-Anne Siegrist, Prof. Laurent Kaiser, Dr Klara Posfay-Barbe

Patients who underwent solid organ transplantation may suffer from mild, significant or major limitations of their immune competence depending upon the time elapsed after transplantation and their immunosuppressive treatment regimen, which depends upon the type of transplant. Consequently, influenza immunization is recommended to all patients with solid organ transplants.

Patients will be recruited in the following groups (N = maximum number):

1. Lung transplant (N=25)
2. Liver transplant (N=50)
3. Kidney transplant (N=100)
4. Pancreas or Langerhans islets +/- kidney transplant (N=50)
5. Heart transplant (N=30)

Inclusion criteria :

1. Patients with solid organ transplants, at least 3 months after transplantation, to whom influenza A H1N1/09 has been prescribed by the physician in charge according to the recommendation of the BAG and who have chosen to be immunized
2. Age ≥ 18 years
3. Followed up in Geneva during the next 3 months
4. Able to sign an informed consent form.

Non-inclusion criteria :

1. Refusal or failure to provide sufficient blood at least for the determination of antibody responses.
2. Transplant recipients after more than 5 years of transplantation, i.e. receiving minimal immunosuppression.
3. Completion of the enrollment phase of the study (cf N max per group).

Sample size

The influence of underlying disease and immunosuppression on vaccine-driven responses to influenza A H1N1/09 is unknown, precluding calculation of the sample size needed to ensure statistical power. A maximal sample size of 200 subjects has been selected based on the number of patients actively followed. This number could be reduced by the interruption of enrolment, should subsequent available evidence indicate that a smaller sample size is sufficient. Note: all patients will receive 2 vaccine doses, allowing direct comparisons between patients of various disease / treatment groups.

Complementary analyses: markers of liver function (ASAT, ALAT, bilirubin, gGT, alkaline phosphatase), of renal function (urea, creatinin), or of pancreatic function (C-peptide) will be assessed as appropriate at the final study visit (4-6 weeks after the last vaccine dose) and compared to baseline values extracted from the medical file (most recent control).

4.5 Pediatric patients (N max = 200)

Remark : this part of the protocol has been submitted to and approved by the Mother-Child Ethical Committee of the HUG (n° CER 09-191).

Main investigator : Prof. Claire-Anne Siegrist

Collaborators : Dr. Klara Posfay-Barbe, Dr Sara Meier, Prof. Laurent Kaiser

In young children, vaccine responses are frequently much weaker than in older individuals. In addition to age, underlying disease and/or treatment may affect immune competence. Children at high risks of complications from influenza A H1N1/09 may thus have a well preserved, limited or significantly depressed immune response capacity: although specific data is not yet available, patients with well-compensated cardiac or pulmonary diseases are expected to raise similar vaccine responses as would healthy children, children with chronic organ diseases that affect their state of health (such as renal insufficiency) could respond less well, whereas immunocompromised patients may succeed or fail to raise vaccine responses depending upon their individual level of immune competence. Consequently, influenza immunization is recommended to all children at high risks of influenza complications because of diseases or treatment affecting their heart, lungs or immune function.

Patients will be recruited in the following groups :

1. Prematurely born infants (≤ 37 weeks GA, currently aged 6 months to 24 months)
2. Children with chronic organ disease that does not affect their general health nor immune competence (e.g. compensated cardiac malformation, asthma that does not require systemic steroid treatment, hemoglobinopathies, etc.)
3. Children with chronic organ disease that affect their general health and may affect their immune competence (e.g. renal insufficiency, cystic fibrosis, chronic liver disease, etc.)
4. Chidren with some degree of immune insufficiency, including HIV-1 infected children, children with cancer, children under immunosuppressive treatment after organ transplant or for autoimmune diseases.

Inclusion criteria :

1. Children at high risks of complications from influenza, to whom influenza A H1N1/09 has been prescribed by the physician in charge according to the recommendation of the BAG and whose parents have accepted immunization
2. Age ≥ 6 months
3. Followed up in Geneva during the next 3 months
4. After signed parental informed consent.

Non-inclusion criteria :

1. Refusal or failure to provide sufficient blood at least for the determination of antibody responses.
2. Completion of the enrollment phase of the study.

Sample size

The influence of underlying disease and immunosuppression on vaccine-driven responses to influenza A H1N1/09 is unknown, precluding calculation of the sample size needed to ensure statistical power. A maximal sample size of 200 subjects is anticipated based on the estimated number of patients. This number could be reduced by the interruption of enrolment, should subsequent available evidence indicate that a smaller sample size is sufficient.

Remark : this part of the protocol has been submitted to and approved by the Mother-Child Ethical Committee of the HUG (n° CER 09-191).

4.6 Healthy subjects to whom immunization is recommended (N max = 250)

Main investigator : Prof. Claire-Anne Siegrist

Collaborators : Prof. Cem Gabay, Prof. Pierre-Yves Dietrich, Prof. Laurent Kaiser, Dr Klara Posfay-Barbe

As influenza is moderately contagious, the risk of contagion 1) within a household, or 2) through health care professionals is markedly higher than through other social contacts. Consequently, household contacts and health care workers constitute a significant risk of transmission to high risk patients, such that their influenza immunization is recommended.

This cohort of healthy individuals will be recruited in the following groups:

1. House-hold contacts of patient with rheumatologic disease (cohort 4.2)
2. House-hold contacts of patient with oncology disease (cohort 4.3)
3. Health care professionals *(only if needed to ensure adequate age comparisons with adult patients)*

Note 1 : as priority influenza A H1N1/09 immunization is not recommended for healthy children, we will not enroll healthy children as controls.

Note 2 : according to the homologation by Swissmedic, healthy adults will only receive 1 vaccine dose.

Inclusion criteria :

1. Household contacts of high-risk patients or health care workers to whom influenza A H1N1/09 has been prescribed according to the recommendation of the BAG and who have chosen to be immunized
2. Age ≥ 18 years
3. Stable in Geneva during the next 3 months
4. Able to sign an informed consent form.

Non-inclusion criteria :

1. Refusal or failure to provide sufficient blood at least for the determination of antibody responses.
2. Completion of the enrollment phase of the study (cf N max).
3. Acute or chronic disease likely to affect vaccine responses.

Sample size

The precise extend / magnitude of vaccine-driven responses to influenza A H1N1/09 is yet unknown, precluding calculation of the exact sample size needed to ensure statistical power. A maximal sample size of 250 subjects has been selected based on similar vaccine studies. This number could be reduced by the interruption of enrolment, should subsequent available evidence indicate that a smaller sample size is sufficient.

5. Study treatment

No drug nor vaccine will be administered for the purpose of this study.

Study participants will have been recommended an influenza A H1N1/09 immunization according to the official recommendations (recommendation for high risk patients: two vaccine doses (i.m.) at a minimal interval of 3 weeks). They will only be recruited after having decided to be immunized.

It is currently planned that Pandemrix® will be used for high-risk adult patients and their close contacts, and Focetria® for high-risk children. The vaccine name will be recorded in the CRF and treated as a variable. The batch number / expiry date will be recorded in the CRF ("page de garde").

Participation to the study will not change the patients' medical care / treatment.

6. Evaluation criteria

Central laboratory: All blood samples will be sent to a central laboratory (the laboratory of the Centre de Vaccinologie, CMU, Centre Médical Universitaire) for processing and serum/PBMC extraction. The samples will be identified by the patient number. Instructions necessary to ensure the appropriate storage and shipment of the sample will be provided on the laboratory form.

B cell immunity: This will be measured on cryopreserved sera, in the Laboratoire de Virologie (HUG). Three co-primary immunogenicity endpoints after vaccination chosen according to international guidelines used to evaluate influenza vaccines are: 1) the proportion of subjects with antibody titers of 1:40 or more on hemagglutination-inhibition assay, 2) the proportion of subjects with either seroconversion or a significant increase in antibody titer, and 3) the factor increase in the geometric mean titer after 2 vaccine doses. These antibody responses will be assessed 1) within 24h of the 1st vaccine dose, for baseline antibodies (as H1N1/09 virus is already circulating in Switzerland, some patients may have been exposed prior to immunization), 2) within 4-6 weeks after the 2nd vaccine dose.

Antibody responses to influenza are best measured by the quantification of functional antibodies. This will be assessed through hemagglutination inhibition (HI) and/or microneutralisation (MN) assays,

using the same endpoints. In both assays, the influenza A H1N1/09 virus is incubated with serial serum dilutions prior to addition to cell cultures (MN) or to erythrocytes (HI). The presence of functional antibodies is reflected in vitro by antibody-mediated cell survival (MN) or protection from viral-induced agglutination (HI). The HI test is simpler, requires less serum volume and is classically used for the determination of influenza immunity. However, it is less sensitive / specific than the MN assay and may generate falsely negative results in young children or immunocompromised patients. Results are expressed as titers (the inverse of the highest dilution still showing a functional effect). An HI titer ≥1:40 correlate best with clinical protection against influenza. It is expected that this will be similar following influenza A H1N1/09. This will be confirmed in the next few months.

Both assays may be generated with frozen serum, allowing the simultaneous evaluation of pre- and post-vaccination samples, thus reducing inter-assay variability. Taken into consideration the need for triplicates, a minimal serum volume of 200ul (0.5ml of blood) will be sufficient for the HI assay. A minimal serum volume of 1ml (2ml of blood) should allow performing both HI and MN assays. This will be specified within the next few weeks and necessary blood volumes reduced to as low values as possible.

T cell immunity: This will be measured on cryopreserved PBMCs, in the laboratories of the Centre de Vaccinologie, CMU. The secondary immunogenicity endpoint was selected as the proportion of subjects with detectable influenza A H1N1/09 specific CD4+ T cell responses after 2 vaccine doses. Indeed, cellular responses may be affected differently than antibody responses by age, underlying diseases and immunosuppression.

To assess vaccine-induced T cell immunity, we will first try to adapt to influenza A H1N1/09 a whole-blood assay measuring the frequency of influenza-specific CD4+ T lymphocytes. Briefly, whole-blood samples will be stimulated in vitro during 6h with influenza A H1N1/09 (or control influenza viruses) in the presence of anti-CD28 and anti CD49d co-stimulating antibodies. Brefeldin A will be added to block extracellular cytokine transport during the last 4h of incubation. Following lysis of red blood cells, lymphocytes will be permeabilised and stained using fluorochrome-conjugated monoclonal antibodies against CD4, CD69 (as activation marker) and IFN-γ or IL-2 prior to assessment of the frequency of influenza-specific activated CD4 T cells by multiparameter flow cytometry. Frequencies of responding cells will be defined as percentages of the total positive events, and considered as reflecting frequencies of influenza-specific CD4 T cells. We will attempt the optimization of this assay, based on our expertise with the detection of varicella-specific vaccine responses, with as little as 3ml of whole blood. Vaccine-induced influenza-specific CD4+ T lymphocytes may however be too few to be identified through a whole blood assay. Should this be the case, we would work with purified peripheral blood mononuclear cells. Briefly, mononuclear cells would be isolated, centrifuged and frozen. After thawing, cells would be restimulated in vitro with influenza A H1N1/09 (or control influenza viruses) prior to fixation, staining and analysis of cytokine producing cells (IFN-g, IL-2, etc.) by flow cytometry. This assay is much more sensitive but requires a higher volume of blood (minimal amount to be defined).

Results will be expressed as numbers of specific cytokine-producing-cells. Direct correlation between cellular responses and protection are not yet established, although these cellular responses are considered as conferring some degree of protection even in the absence of neutralizing antibodies.

The minimal volume of blood required for the analysis of cellular responses will be defined during the next few weeks. Blood sampling will be limited by the age of the patient (total of 5ml in young children, 10 ml in older children, 30ml in adults). Thus, T cell responses will only be determined in patients for whom enough blood will have been harvested.

Vaccine-associated adverse events:

Studying vaccine reactogenicity / safety is not an objective of this study. However, any vaccine study is expected to offer the follow-up of potential adverse events to study participants. Parents or patients will be invited to record on a diary card temperature, potential local or systemic reactions to each vaccine dose as well as any unexpected event. They will be offered medical advice if in doubt concerning the occurrence of a potential vaccine-associated adverse event. It is expected that immunosuppressed patients will raise weaker inflammatory responses than healthy adults. As inflammatory reactions reflect innate responses and shape adaptative, B and T cell immunity, we will seek for potential correlations between inflammatory and immune responses.

All solicited local adverse events will be considered as related to the H1N1 vaccine, whereas the clinical investigators will assess the causality of solicited or unsolicited systemic adverse events.

Underlying disease and treatment:

Information on the underlying disease of these high risk patients will be collected through a medical history and by assessing their medical file. Data will include demographic, clinical characteristics, with a special focus on the degree of immunocompetence, and treatment at time of immunization.

To more objectively assess any potential changes in underlying disease, blood markers of underlying disease activity / graft function will be assessed at the final study visit and compared to baseline values.

7. Data collection and management

Source of data: Information relative to the patient underlying disease and/or treatment will be retrieved through a medical history taken at time of enrollment and completed by access to the patient medical record – as needed. Paper-based CRF will be considered as source data.

CRF: A paper-based CRF will be used to facilitate enrollment and data collection at several locations within the HUG. Subjects will be identified on study documents by the first two letters of their first and last names and by a study number. Subjects will not be identified by name on any study document.

This CRF will include common items to all participants as well as cohort-specific items. For each specific cohort, only the common and cohort-specific items will be included in the printed paper-based CRF, ensuring that inappropriate items are not included. All CRF data will be entered legibly in black ink. Amendments and errors on the CRF will not be erased, covered with correction fluid or completely crossed-out; rather a single line will be drawn through the error and the correction will be initialled and dated by the investigator or authorized personnel. CRFs will be currently maintained, reviewed, clarified and processed in accordance with the Standard Operating Procedures and working practices.

The CRF has now been generated by clinical research professionals (Electricpaper S.A., http://www.electricpaper.de/produkte/teleform.html) so as to allow processing by the Cardiff Teleform Intelligent Document Capture and Processing system (http://www.cardiff.com/products/teleform/), ensuring their adequate transfer into a single common ACCESS data base. The database has also been constructed by Electricpaper S.A., ensuring its adequacy with the CRFs.

Documents storage and keeping: All documents, including the original study protocol, notification letters from the IRB and Swissmedic, paper-based CRFs will be kept secure in accordance with the good clinical practice guidelines and applicable regulatory requirements.

8. Adverse events management

No drug nor vaccine will be administered for the purpose of this study.

However, study participants will have been recommended by their physician an influenza A H1N1/09 immunization with a licensed influenza A H1N1/09 vaccine. Any potential serious vaccine-associated adverse event will be notified by the physician in charge of the patient, according to Swissmedic's instructions. Should the study investigators be made aware of a missing notification, they would report it immediately and follow Swissmedic instructions.

To note: the immunization of high-risk patients will be taking place throughout Switzerland (and many other countries) at the same time. Vaccine-safety monitoring will thus be ongoing at a national and international level, providing information on potential vaccine-safety signals.

DEFINITION

An adverse event (AE) is defined as any adverse change from the patient's baseline condition. An adverse event does not necessarily have a causal relationship with the study treatment. A detailed description of any solicited adverse event will be entered in the CRF, including its intensity graded on a 6-point scale (mild (1-2), moderate (3-4), or severe (5-6)), the action undertaken for this event, the investigator's assessment of the relationship of the event to treatment, and the outcome. All serious

adverse events encountered during the clinical trial will be followed up until the event is either resolved or adequately explained.

A serious adverse event (SAE) as defined by ICH is any untoward medical occurrence that at any dose meets any of the following conditions:

- Results in death
- Is life-threatening (the subject was at risk of death at the time of the event. It does not refer to an event which hypothetically might have caused death if it were more severe)
- Requires or prolongs hospital stay significantly
- Results in persistent or significant disability/incapacity,
- Constitutes a congenital anomaly/birth defect
- Is an important medical event

Important medical events are those that may not be immediately life threatening, but are clearly of major clinical relevance. They may jeopardize the subject, and may require an intervention to prevent one of the other serious outcomes noted above.

The nature of the SAE (expected or unexpected) as well as the relationship between the SAE and the study drug (imputability) will we assessed in order to define whether it is a Suspected Unexpected Serious Adverse Reaction (SUSAR) or not. Reports of all SAE will be submitted to the Ethics Committee and SwissMedic within 15 days for the SUSAR (as soon as possible for a death and within 7 days). The non-SUSAR SAE will be reported by the way of annual reports.

9. Quality control and assurance

This study will be performed according to GCP guidelines through a joint effort of senior investigators with extensive experience working with high risks patients, carrying out clinical investigations, working with influenza viruses and/or assessing complex vaccine responses.

Patients will be recruited at time of a routine medical visit in the respective Divisions of the Geneva University Hospital, by their physicians. The physicians will be instructed to provide adequate information and explanation regarding the aim, procedure and potential benefits and side effects of the study to their patients.

Professional expertise has been secured to prepare the CRF and the related data base. A data manager will be responsible for electronic data transfer and handling of the data base. Blood samples

will be handled by fully trained laboratory technicians. Vaccine responses will be assessed by virology and immunology experts of the Laboratoire de Vaccinologie and the Centre de Vaccinologie et d'Immunologie Néonatale, under appropriate biosafety conditions.

An expert biostatistician (Dr Combescure) will be responsible for all statistical analyses. All investigators will contribute to the analysis and interpretation of the data, and to their publications.

<u>Reporting Procedures</u>

- Serious adverse events, see above
- Amendments to the protocol. Minor amendments (typographical errors, changes in administrative procedures and the like) will be bundled and submitted to the Ethics Committee at regular intervals of three months. Substantive changes will be formulated as study amendments and submitted to the Ethics Committee, and to Swissmedic, following the legal procedures as outlined on the Swissmedic website.
- Reports. The study duration for the participants is 12 weeks, but data generation and interpretation will be long. We expect the study to be terminated within 2 years. If this is not the case, and intermediate report will be produced and transmitted to the Ethics Committee and Swissmedic one year after the first patient starts the study. If the expected delays are kept, a Final Report will be produced two years after the study start.

Clinical Site Monitoring/Audit

Personal medical data may be reviewed at clinical sites by properly authorised individuals as part of monitoring or audit of the trial, but such information will be treated as strictly confidential and will in no circumstances be made publicly available.

10. Statistics

10.1 <u>Number of subjects</u>

A maximum of 1500 subjects (including 250 controls) will be enrolled in 6 parallel cohorts including patients with HIV-1 infection (n=200), rheumatologic disorders (n=300), malignancies (n=300), solid organ transplant recipients (n=250), high-risk children (n=200) and healthy contacts (n=250).

The expected proportion of responder in the control group is 83% [ref NEJM] after 1 dose of influenza A H1N1/09-MF59 adjuvanted vaccine. With 250 controls, assuming a bilateral risk of 5%, we can expect to assess the proportion of 83% with a 95% confidence interval [78.3-87.3]. From [Clark W, 2009], the expected response rate is 91% after 2 doses of non-adjuvanted vaccine. The sample size of 250 leads to an expected confidence interval of [87.0-95.0]. If 2 doses of adjuvanted vaccines elicit

seroprotection in a higher proportion than non-adjuvanted vaccines, the power of the study will be increased further.

The expected confidence interval was also assessed for the sample size of the different groups and for various response rates:

		Sample size			
		200	225	250	300
Expected response rate	90%	[85.8-94.2]	[86.1-93.9]	[86.3-93.7]	[86.6-93.4]
	85%	[80.0-90.0]	[80.3-89.7]	[80.6-89.4]	[81.0-89.0]
	80%	[74.4-85.6]	[74.8-85.2]	[75.0-85.0]	[75.5-84.5]
	75%	[69.0-81.0]	[69.3-80.7]	[69.6-80.4]	[70.1-79.9]
	70%	[63.6-76.4]	[64.0-76.0]	[64.3-75.7]	[64.8-75.2]

We expect a difference in the percentage of responders between the control group and the other groups, but the magnitude of this difference is presently unknown. Given the sample size, we assessed the statistical power of the comparison of the response rates, assuming a response rate of 83% in the control group [Clark W, 2009], and 68%, 73%, and 78% in the groups of patients (corresponding to differences of 15%, 10% and 5% respectively). The risk α was 5% (two-sided).

The results are presented in the following table.

Group	Expected response rate in the group	Expected response rate in controls	Statistical power
patients with HIV-1 infection (n=200)	68%	83%	95%
	73%	83%	70%
	78%	83%	25%
rheumatologic disorders (n=250)	68%	83%	97%
	73%	83%	74%
	78%	83%	25%
malignancies (n=300)	68%	83%	98%
	73%	83%	79%
	78%	83%	29%
solid organ transplant recipients (n=225)	68%	83%	96%
	73%	83%	73%
	78%	83%	26%

high-risk children (n=200)	68%	83%	95%
	73%	83%	70%
	78%	83%	25%
healthy contacts (n=250)	68%	83%	97%
	73%	83%	74%
	78%	83%	25%

Overall, the planned sample size will provide a statistical power ranging from 70% to 79% for an expected difference of 10% in the response rate. The power is systematically beyond 95% for an expected difference of 15%. Given that many patients are markedly immunosuppressed, we do expect that differences will indeed be higher than 15%.

Assuming a response rate of 91% (after 2 doses of unadjuvanted vaccine), and differences of 15%, 10% and 5%, the power will be that described in the following table :

Group	Expected Response rate in the group	Expected response rate in controls	Statistical power
patients with HIV-1 infection (n=200)	76%	91%	99%
	81%	91%	85%
	86%	91%	36%
rheumatologic disorders (n=250)	76%	91%	99%
	81%	91%	88%
	86%	91%	37%
malignancies (n=300)	76%	91%	99%
	81%	91%	91%
	86%	91%	42%
solid organ transplant recipients (n=225)	76%	91%	99%
	81%	91%	87%
	86%	91%	37%
high-risk children (n=200)	76%	91%	99%
	81%	91%	85%
	86%	91%	36%
healthy contacts (n=250)	76%	91%	99%
	81%	91%	88%
	86%	91%	37%

The planned sample size will provide a statistical power ranging from 85% to 91% for an expected difference of 10% in the response rate. Given that many patients are markedly immunosuppressed, we do expect that differences will be higher than 10%.

10.2 Data analysis

The general objective of this parallel-cohort study is to provide information on determinants that affect vaccine responses:

- in each cohort (i.e. HIV, rheumatology, oncology, transplant, pediatric, healthy controls), e.g. influence of CD4 T cell count among HIV-1 patients, of immunosuppressive regimen among patients with rheumatologic disorders, of time elapsed after liver transplant, etc.
- as compared to healthy individuals
- across the various cohorts – allowing to progress towards the identification of makers of immune competence.

Primary analyses: The analyses will be descriptive, consisting of an assessment of each primary endpoint (*proportion with titers ≥ 1:40, proportion with seroconversion or significant increase in antibody titer, mean geometric titers - as assessed by hemagglutination inhibition*) with their confidence bounds (calculated by the exact method of Clopper-Pearson):

- for each cohort (i.e. HIV, rheumatology, oncology, transplant, pediatric, healthy controls)
- for each group with each cohort (e.g. HIV-infected patients with CD4 >500 or < 350; liver versus kidney transplant, etc.)
- for each sub-group (defined by potentially relevant variables) within a group, a cohort and across cohorts.

Proportions and titers will be compared (by Chi-square or Fisher exact test, and Wilcoxon's test or Kruskal-Wallis test) between the groups and the controls.

The influence of potentially relevant variables will be tested in an univariate way. A logistic model will then be constructed to study the factors likely to explain the proportion reaching the primary endpoints: this will include the subgroups plus the variables with an effect in the previous analysis. An interaction between the subgroups and other factors will be considered if necessary. A multivariate analysis (logistic regression) will be performed to compare high-risk patients and healthy controls, while adjusting for other variables. The goodness-of-fit will be analysed using the Hosmer-Lemeshow' test.

Secondary analyses: The same approach will be applied to the quantification of neutralizing antibodies. For CD4 T cell responses endpoints, whenever available, the comparison will be performed using a non parametric test (Wilcoxon's test or Kruskall-Wallis test). A multivariate analysis

will be also performed. For this purpose, we will transform the CD4 T to get a gaussian distribution. The quality of model will be analysed (R-square, interpretation of residuals).

Recorded solicited local and systemic adverse events during 7 days after each vaccination will be described and compared using non-parametric methods. Similarly, changes in markers of underlying disease activity / graft function will be described and compared across study groups.
All the tests will be two-sided with a significance level at 5%.

Addendum: given the good immunogenicity of adjuvanted vaccines, Swissmedic has defined that a single vaccine dose will be sufficient for healthy controls. Thus, we will assess which are the determinants that allow 2 vaccines doses in immunosuppressed patients to raise responses similar to one dose in healthy adults. Comparison of responses to the first vaccine dose will be assessed in the rheumatology and oncology cohorts, where patients will be asked to provide an extra sample. Although this will be optional, we expect most patients to accept this procedure, a routine in their treatment.

10.3 Dropouts and missing data management

The per-protocol immunogenicity analyses will only include subjects with available blood samples before and after immunization – and include a complete set of analyses.
The intention-to-treat analyses will include all subjects who provided a post-immunization blood sample – even in the absence of a pre-immunization sample. The pre-post immunization comparisons will not be possible and the interpretation of vaccine responses will have to take into consideration the likelihood of pre-immunization exposure to influenza A H1N1/09.
Subjects dropping out of the study prior to the post-immunization blood sampling will not be included in the statistical analyses. Patients will be replaced if there is no vaccination.

Reasonable attempts will be made to limit the amount of missing data related to serious adverse event and patient outcomes to ensure that important information related to the primary objective of the study is captured. In case of missing data, we will first try to retrieve the information in the patient databases of the University Hospital of Geneva. No imputation will be made on the CRF for missing data and further details of how missing data has been handled will be outlined in the study analysis plan. Variables with less than 20% of missing data will be kept in the analyses, and the patients with missing data will be excluded from the multivariate analyses. Variables containing more than 20% of missing data will not be included in the multivariate analyses and a logistic regression model will be performed to identify the factors associated with missing data.

10.4 Criteria for the termination of the trial

The trial will be terminated whenever the maximal sample size has been reached or whenever the available evidence indicates that increasing the sample size would not improve statistical significance any further.

11. Ethical considerations

11.1 Risks and benefits

The only risks associated to this study are those of venous puncture (hematoma, etc.).
The potential benefits include the identification of patients who succeeded or failed to generate vaccine responses to influenza A H1N1/09 vaccines. This information will provide relief to responding high-risk patients (or their parents) and identify non-responding patients for whom strict prophylactic / therapeutic measures would remain required despite immunization. Thus, this study confers direct benefits to the participants.

In addition, it will provide most needed information on the protection that adjuvanted influenza vaccines may offer to high-risk patients with underlying diseases and various levels of immune competence. This is an important indirect benefit for all high-risks patients.

11.2 Ethical approval

This study was submitted and approved by the relevant institution Ethical Committee (Comité d'Ethique de Médecine interne, Comité d'Ethique Mère-Enfant).
It will not start before official approval by Swissmedic.

11.3 Information and consent

Written consent will be obtained from all patients (or from parents or legal guardians, and children whenever possible) before any study procedure (i.e before the first blood sample).

11.4 Confidentiality

All records will be stored in secure files and computers. Data will be anonymized for analysis and publication. Only the investigators and legal authorities will have access to the data.

12. Publication policy

This study will be registered in the NIH international registry of clinical trials. It is intended for (several) publications in scientific journals with peer-reviewed editorial policy. Study results will be shared as confidential privileged information with Swissmedic and the Federal Office of Public health at time of manuscript approval.

Individual patient results will be communicated to the physician in charge.

13. Financing and insurance

This study will be run independently from the pharmaceutical industry, so as to increase its perceived credibility.

Patient enrolment, data collection of underlying disease / treatment and pre-immunization venous sampling will be supported by existing resources from the investigators of each specific cohort. The costs of data acquisition (CRF), data management and data analyses as well as vaccine response analyses will be supported by existing research funds of the Center for Vaccinology and Neonatal Immunology (Prof. Claire-Anne Siegrist) and by an advanced researcher research grant submitted to the Centre de Recherche Clinique. Additional research funds will be seeked by the investigators to support the determination of T cell responses in as many participants as possible.

There will be no financial incentive to patients or participating physicians, to whom the (expensive) determination of vaccine responses will be offered free of charge. To note, the costs of influenza A H1N1 immunization will be free in Switzerland for all high-risk patients and their contacts, with no franchise nor participation (quote-part).

The study participants will be covered against the risks associated to this study by an insurance issued to the Hôpitaux Universitaires de Genève.

14. 2010 study extension: immune memory

Although analyses are ongoing, the follow-up of 766 immunocompromised adult patients and 133 controls demonstrated that :

- adjuvanted vaccines were well tolerated, inflammatory reactions being fewer / less intense in immunocompromised patients than in healthy controls and severe adverse events being essentially non-associated to immunization (see interim report in Annex); immunization did not trigger autoimmune disease exacerbations nor graft rejection and did not require the intensification of immunosuppressive treatments. A transient increase of HIV-1 viremia was noted, as previously described following seasonal influenza immunization, but remained without clinical influence.
- adjuvanted vaccines elicited potent antibody responses after a single vaccine dose in healthy individuals; similar responses were observed after 2 doses in HIV-infected patients; however, vaccine responses remained significantly lower in transplant patients.

In October 2010, seasonal influenza immunization will be recommended to these high-risk patients. They will receive a non-adjuvanted vaccine including 3 influenza strains: the same A/H1N1/09 strain as in 2009, and 2 new strains (A/H3N2/10 et B).

We would thus want to extend our observation by the assessment of responses to the seasonal influenza vaccine in patients with altered (transplant), partial (HIV-infection) or normal (healthy controls) immune competence. We plan to measure how age, underlying disease and immunosuppression influences:

1. the induction of immune memory, measured :
    a. by the persistence of antibodies elicited in 2009 by the influenza A/H1N1/09 adjuvanted vaccine, compared to non-adjuvanted vaccines against 2009 seasonal influenza
    b. by the booster effect of the influenza A/H1N1/09 strain included in the 2010 vaccine, compared to the primary responses elicited by the A/H3N2/10 strain.
2. the influence of non-adjuvanted vaccines against seasonal influenza 2010 on HIV-viremia, compared to the influence of the 2009 adjuvanted vaccines.

Design of the extension phase:

First visit : (at time of seasonal influenza immunization): venous bleeding for the quantification of baseline antibody titers against influenza A/H1N1/09 and seasonal viruses (volume 2ml); medical history. HIV-infected patients VIH : viremia / molecular analyses (volume 7 ml), CD4 lymphocytes (volume 3ml).

Second visit : (4-6 weeks later) : venous bleeding for the quantification of vaccine-induced responses to influenza A/H1N1/09 and seasonal viruses ; collection of adverse events. HIV-infected patients : viremia / molecular analyses (volume 7 ml)

Note : HIV-infected patients with increased viremia would be invited to return for a medical visit 3 months after immunization to confirm that viral control is appropriate .

Antibody responses (and HIV-RNA viral load) will be measured and compared as previously described.

We will use the same CRF / carnet des vaccinations to obtain an up-to-date medical history and follow-up potential vaccine adverse events.

Inclusion criteria :

1) Adult patients with HIV-infection, after solid organ transplant or healthy (for example health care professionals) to whom 2010 seasonal influenza immunization is recommended
2) Who were immunized against influenza A/H1N1 in the fall of 2009
3) Who are willing to return for a second visit 30 days later
4) Who have signed an informed consent.

Exclusion criteria :

1) Refusal or failure to provide blood (minimum volume 2 ml).

Risks and benefits

Risks : Those of venous bleeding: bruising at the site of injection may not be excluded.

Indirect benefits: Improvement of the knowledge, allowing defining whether / which immunocompromised patients should receive adjuvanted seasonal influenza vaccines in the future.

Statistical evaluation

This will be a descriptive analysis of the influence of age and immune suppression on the induction of immune memory by adjuvanted versus non-adjuvanted influenza vaccines. This influence is yet unknown, precluding sample size estimation. However, data collected in 2009 confirmed that the inclusion of approximately 150 patients by group was sufficient to identify significant differences between healthy and immunosuppressed patients.

Analyses will be performed as previously described and found appropriate.

Funding

The costs of this study extension will be covered by existing research funds and new sources of funding (pending demand to the Swiss HIV Cohort Study).

Ethical approval

This extension should ideally start on October 1st (influenza immunization campaign), but will not be initiated prior to its approval by the Ethical Committee and Swissmedic.

15. Annexes

- Annex 1 : Information to study participants or their parents / legal guardians

  This includes the information to study participants (3 forms):

  1. Adult patients
  2. Pediatric patients *(this form was approved by the CE Mère Enfant, n° CER 09-191)*
  3. Healthy contacts of high-risk patients

- Annex 2 : Consent forms

  This annex includes the consent forms (2 forms):

  1. Adult patients and controls
  2. Pediatric patients *(this form was approved by the CE Mère Enfant, n° CER 09-191)*

- Annex 3 : List of variables for inclusion into the CRF / database

  Note : A paper-based CRF is now included - generated by Electricpaper S.A., http://www.electricpaper.de/produkte/teleform.html).

- Annex 4 : "Carnet des vaccinations"

  This will be given to the participants to record potential solicited adverse events.

- Annex 5 : Interim safety report
